# Kombucha Fermentation Remodels the Phytochemical Composition of Gooseberry Juices and Reveals a Divergence Between Phenolic Abundance and HepG2 Cell Growth Inhibition

**DOI:** 10.3390/ijms27219794

**Published:** 2026-10-31

**Authors:** Akshay K. Chandran, Gloria-Renate Klein, Joanna Kawa-Rygielska, Anita Rywińska, Antoni Szumny, Jiaxi Zhang, Huiying A. Zhang, Cayla Boycott, Barbara Stefanska, Joanna Kolniak-Ostek

**Affiliations:** 1 Department of Fruit, Vegetable and Plant Nutraceutical Technology, Wrocław University of Environmental and Life Sciences, 37 Chelmonskiego Street, 51-630 Wroclaw, Poland; akshay.chandran@upwr.edu.pl; 2 Food, Nutrition and Health Program, Faculty of Land and Food Systems, The University of British Columbia, 2205 East Mall, Vancouver, BC V6T 1Z4, Canada; gloklei@student.ubc.ca (G.-R.K.); aaaaalice5423@gmail.com (J.Z.); amelie.huiying.zhang@ubc.ca (H.A.Z.); caylasboycott@gmail.com (C.B.); 3 Department of Fermentation and Cereals Technology, Wrocław University of Environmental and Life Sciences, 37 Chelmonskiego Street, 51-630 Wroclaw, Poland; joanna.kawa-rygielska@upwr.edu.pl; 4 Department of Biotechnology and Food Microbiology, Wrocław University of Environmental and Life Sciences, 37 Chelmonskiego Street, 51-630 Wroclaw, Poland; anita.rywinska@upwr.edu.pl; 5 Department of Food Chemistry and Biocatalysis, Wrocław University of Environmental and Life Sciences, 27 Norwida Street, 50-375 Wroclaw, Poland; antoni.szumny@upwr.edu.pl

**Keywords:** *Ribes uva-crispa*, SCOBY fermentation, polyphenols, phytochemical transformation, HepG2 cells, gene expression

## Abstract

Kombucha fermentation can modify the phytochemical composition and biological properties of fruit-based beverages, although these changes may differ depending on the fermentation substrate. This study investigated the effects of 10-day kombucha fermentation using a symbiotic culture of bacteria and yeast (SCOBY) on red and white gooseberry (*Ribes uva-crispa*) juices supplemented with sucrose or buckwheat honey. Physicochemical characteristics, phenolic and volatile profiles, antioxidant capacity, enzyme inhibitory activities, HepG2 cell growth, gene expression, and sensory acceptance were evaluated. Fermentation decreased sugar concentrations and increased organic acid and ethanol contents. The concentrations of several flavan-3-ols, including epigallocatechin and epigallocatechin gallate, increased, whereas selected oligomeric procyanidins decreased. Red gooseberry beverages were characterized by a greater contribution of esters to their volatile profiles, while alcohols predominated among the identified volatile compounds in white gooseberry beverages. Red gooseberry formulations generally exhibited higher phenolic concentrations and antioxidant capacity, with the honey-supplemented variant showing particularly high values. However, phenolic-enriched extracts isolated from fermented white gooseberry beverages inhibited HepG2 cell growth more strongly than extracts from red gooseberry beverages. White gooseberry extracts also altered the expression of selected genes associated with metabolism and cancer-related signaling. Sensory evaluation indicated the highest overall acceptance for sucrose-supplemented red gooseberry juice. These findings demonstrate that the effects of kombucha fermentation depend on gooseberry juice type and carbon source, and that phenolic abundance and antioxidant capacity do not necessarily predict cellular growth-inhibitory effects or sensory acceptance.

## 1. Introduction

Fermentation using symbiotic cultures of bacteria and yeast (SCOBY) has emerged as an effective strategy for improving the functional properties of plant-based beverages through the microbial biotransformation of bioactive compounds [1,2]. Kombucha-type fermentation modifies polyphenolic profiles, increases the availability of low-molecular-weight phenolics, and generates metabolites with improved biological activity [2,3]. These transformations are particularly relevant to functional foods because the biological properties of plant-derived beverages may depend not only on the overall abundance of phytochemicals, but also on their qualitative composition and fermentation-induced conversion.

European gooseberry (*Ribes uva-crispa*) is a valuable but underexplored substrate for such processes. Gooseberries are rich in polyphenols, vitamin C, and other phytochemicals [4], while red cultivars generally contain higher levels of phenolic compounds and exhibit greater antioxidant capacity than white cultivars [5]. This natural compositional contrast provides an opportunity to investigate whether differences in phytochemical abundance are maintained after fermentation and whether they are reflected in biological activity. Importantly, processing can substantially modify the composition and properties of plant phenolics through processes such as depolymerization, deglycosylation, and the release of bound compounds [2,6].

Although SCOBY fermentation has been extensively investigated in tea-based systems and has increasingly been applied to fruit-derived matrices [7,8], limited information is available regarding its application to gooseberry juice. In particular, little is known about how the contrasting phytochemical composition of red and white gooseberries influences fermentation-induced changes or how these effects are modified by the choice of carbon source, such as sucrose or honey. More importantly, the extent to which fermentation-driven compositional changes correspond to biological responses remains insufficiently understood, especially when conventional measures such as total polyphenol content and antioxidant capacity are compared with effects observed at the cellular level.

The selection of sucrose and buckwheat honey as alternative carbon sources was intended to examine whether the composition of the fermentation substrate influences phytochemical transformations and the functional characteristics of gooseberry-based kombucha. Sucrose was selected as a conventional carbohydrate source commonly used in kombucha production, providing a reference formulation for comparison. In contrast, buckwheat honey represents a more compositionally complex substrate containing fermentable sugars together with minor constituents, including phenolic compounds, organic acids, and minerals, which may influence fermentation processes and the properties of the resulting beverages. Comparing these two carbon sources in red and white gooseberry juices enabled the evaluation of whether the effects of sweetener selection differed between fruit matrices with contrasting phytochemical characteristics. Accordingly, the four formulations constituted a two-factor experimental design involving gooseberry juice type (red or white) and carbon source (sucrose or buckwheat honey).

Therefore, this study investigated the effects of SCOBY fermentation on the physicochemical characteristics, polyphenolic and volatile profiles, and biological properties of red and white gooseberry juices supplemented with either sucrose or honey. We hypothesized that fermentation would induce matrix-dependent changes in phytochemical composition and that the resulting biological activity would not necessarily be proportional to total polyphenol abundance. To address this hypothesis, compositional changes were evaluated alongside antioxidant and enzyme-inhibitory activities, HepG2 cell growth, and the expression of selected genes involved in metabolic and oncogenic signaling. This integrated approach was used to examine how fermentation-induced phytochemical remodeling relates to the functional properties of gooseberry-based fermented beverages.

## 2. Results and Discussion

### 2.1. Changes in Culturable Microbial Populations During Fermentation

Microbial populations in gooseberry-based kombucha changed markedly during fermentation, with the magnitude of these changes differing among microbial groups and juice formulations (Table 1). Total viable counts (TVC) increased between the initial sampling point and day 10 in all beverages. The increase was particularly pronounced in RGJ-S, from 6.0 × 10^3^ to 4.1 × 10^4^ CFU/mL, and in RGJ-H, from 3.2 × 10^3^ to 4.0 × 10^4^ CFU/mL. Lower final TVC values were observed in the white gooseberry variants, reaching 1.0 × 10^3^ and 7.3 × 10^3^ CFU/mL in WGJ-S and WGJ-H, respectively.

Presumptive lactic acid bacteria (LAB) showed the most pronounced increase during fermentation. Their initial counts were below 10^2^ CFU/mL in all formulations, whereas after 10 days they reached 4.4 × 10^5^ and 3.5 × 10^5^ CFU/mL in RGJ-S and RGJ-H, respectively. Lower final counts were observed in WGJ-S (1.9 × 10^4^ CFU/mL) and WGJ-H (4.2 × 10^4^ CFU/mL), indicating a marked difference between the red and white gooseberry matrices. In contrast, presumptive acetic acid bacteria (AAB) remained below 10^2^ CFU/mL in most samples. Detectable counts were observed only in RGJ-S (1.0 × 10^2^ CFU/mL) and WGJ-S (3.9 × 10^2^ CFU/mL) after 10 days of fermentation. During fermentation, visible growth of the initially introduced SCOBY was observed. Additionally, a new SCOBY-like pellicle initially formed at the air–liquid interface and subsequently descended towards the bottom of the fermentation vessel.

This profile differs from that commonly reported for conventional tea-based kombucha, in which AAB constitute an important component of the fermentation microbiota [7,9]. The low recoverable AAB counts observed in the present study may indicate that the gooseberry juice matrix and/or the applied fermentation conditions were less favorable for their proliferation. The relatively low counts of culturable acetic acid bacteria, together with the substantial ethanol concentrations detected after 10 days of fermentation, suggest that alcoholic fermentation was an important component of the process. At the same time, the depletion of fermentable sugars, accumulation of organic acids, and decrease in pH demonstrate that the inoculated gooseberry juices underwent substantial biochemical changes during fermentation. Although a new pellicle was observed, its formation alone cannot confirm the identity or metabolic activity of the microorganisms involved. Furthermore, culture-dependent enumeration reflects only microorganisms recoverable under the applied cultivation conditions and cannot establish the total abundance or metabolic activity of acetic acid bacteria. Therefore, while the results confirm fermentation and acidification of the SCOBY-inoculated juices, they do not allow the relative contributions of yeasts, acetic acid bacteria, and other microorganisms to be conclusively determined.

Yeast and filamentous fungal counts also increased substantially during fermentation. Initial counts ranged from 2.5 × 10^3^ to 6.0 × 10^3^ CFU/mL and increased to 3.5 × 10^3^–1.6 × 10^5^ CFU/mL after 10 days. The largest increases were observed in both RGJ formulations, reaching 1.6 × 10^5^ and 1.5 × 10^5^ CFU/mL in RGJ-S and RGJ-H, respectively, whereas WGJ-H reached a similarly high final count of 1.6 × 10^5^ CFU/mL. These results demonstrate that microbial development depended on both the microbial group and beverage formulation, consistent with the substrate-dependent variability previously reported for kombucha beverages [10].

The culture-dependent analysis indicated distinct changes in recoverable microbial populations during gooseberry juice fermentation, with the most pronounced numerical differences between red and white gooseberry beverages observed for presumptive LAB. The consistently low recoverable AAB counts indicate a microbial profile different from that typically associated with tea-based kombucha. These observations provide a microbiological context for the changes in substrate utilization and metabolite formation described in the following sections.

Previous studies have demonstrated that microbial population changes during kombucha fermentation are associated with changes in the chemical composition of the beverage. Klawpiyapamornkun et al. [7], who investigated kombucha supplemented with Indian gooseberry, monitored bacterial and yeast populations at three-day intervals alongside physicochemical changes, illustrating the value of intermediate microbiological sampling. Furthermore, Wang et al. [9] demonstrated that interactions between yeasts and acetic acid bacteria in a defined microbial consortium influence organic acid production and ethanol metabolism. However, these findings cannot be directly extrapolated to the present gooseberry-based beverages because of differences in substrate composition and microbial communities. In particular, the low recoverable counts of presumptive acetic acid bacteria in our study do not allow their contribution to acid formation to be established.

The differences in culturable microbial populations between the gooseberry formulations suggest that the fruit matrix and carbon source may have influenced microbial growth during kombucha fermentation. In particular, the substantially higher final counts of presumptive lactic acid bacteria in red gooseberry beverages compared with white gooseberry beverages indicate that the two juice matrices provided different conditions for microbial proliferation. However, the present culture-dependent measurements do not allow the specific factors responsible for these differences to be identified. The increase in yeast and filamentous fungal counts was accompanied by sugar depletion and ethanol accumulation, consistent with fermentative activity, although these measurements do not establish a direct quantitative relationship between microbial abundance and metabolite formation. Similarly, the low recoverable counts of presumptive acetic acid bacteria should be interpreted cautiously in view of the accumulation of acetic acid during fermentation, since plate counts may not fully represent microbial activity or the composition of the fermentation community. An important limitation of the study is that microbial enumeration was performed only at the beginning and end of fermentation. Consequently, changes in microbial populations at day 5, when substantial physicochemical and phytochemical transformations were already evident, could not be directly assessed. Future studies incorporating intermediate sampling points and culture-independent microbial profiling would provide a more complete understanding of microbial succession and its relationship with the chemical transformations observed in gooseberry-based kombucha.

### 2.2. Chemical Changes During Fermentation

SCOBY fermentation substantially modified the physicochemical and chemical characteristics of gooseberry-based beverages, with the magnitude of these changes depending on juice type, carbon source, and fermentation time (Table 2). Total soluble solids decreased progressively in all formulations, from initial values of 10.5–14.4 °Brix to 5.8–9.1 °Brix after 10 days of fermentation. This decrease was accompanied by substantial changes in the sugar profile, ethanol accumulation, and organic acid formation, reflecting progressive conversion of the fermentation substrates.

Sugar utilization differed markedly between sucrose- and honey-supplemented beverages. In the sucrose-supplemented variants, sucrose decreased from approximately 890 mg/100 mL at D_0 to 98.8 and 120.5 mg/100 mL at D_10 in WGJ-S and RGJ-S, respectively. Fructose and glucose concentrations also declined substantially during fermentation. In honey-supplemented beverages, the initial sucrose concentration was considerably lower and decreased to 2.4 mg/100 mL in WGJ-H and below the quantifiable level in RGJ-H by D_10, while marked depletion of fructose and glucose was also observed. These differences are consistent with the distinct initial carbohydrate composition of sucrose- and honey-supplemented matrices and may have influenced substrate utilization during fermentation [11].

Ethanol accumulated progressively in all beverages, although its final concentration differed among formulations. After 10 days, the highest concentration was observed in WGJ-S (6.28%), followed by WGJ-H (4.76%) and both RGJ variants (4.27%). Ethanol formation accompanied the pronounced depletion of fermentable sugars and is consistent with yeast activity during fermentation. Ethanol accumulation has also been reported in fruit-based kombucha beverages and is strongly dependent on substrate composition and fermentation conditions [7,12]. Organic acid profiles also changed substantially during fermentation. Lactic acid increased from approximately 0.01 g/100 mL at D_0 to 0.66–0.72 g/100 mL at D_10, whereas malic acid showed a more moderate increase, reaching 1.52–1.78 g/100 mL. Acetic acid, which was not detected at D_0 or D_5, reached 2.20–3.03 g/100 mL at D_10. This accumulation occurred despite the low recoverable counts of presumptive AAB observed in the culture-dependent microbiological analysis, indicating that microbial counts and metabolite accumulation did not follow a simple proportional relationship. Glucuronic acid was not detected under the applied analytical conditions, despite being reported as a metabolite of conventional kombucha fermentation [13].

The simultaneous depletion of fermentable sugars and accumulation of ethanol and organic acids are consistent with the complementary metabolic activities typically associated with kombucha fermentation. Yeasts can contribute to sugar conversion and ethanol formation, whereas bacterial metabolism may contribute to the accumulation of organic acids. Nevertheless, the relative contribution of individual microbial groups cannot be determined from the present culture-dependent enumeration data. Notably, the relatively narrow range of final pH values did not correspond to identical organic acid concentrations across the formulations. This observation suggests that pH alone is insufficient to characterize the extent of fermentation-induced acidification, as it also depends on the composition and buffering properties of the beverage matrix.

Fermentation was additionally accompanied by a decrease in pH and dry matter, whereas changes in viscosity depended on the beverage formulation and fermentation stage. The final pH values converged within a relatively narrow range of 2.73–2.82 despite differences in their initial values. Dry matter decreased in all formulations, with the largest numerical reductions observed in WGJ-S and RGJ-H. These physicochemical changes, together with sugar depletion and ethanol and organic acid formation, demonstrate extensive transformation of the gooseberry juice matrices during fermentation. Similar substrate-dependent physicochemical changes have been reported in other kombucha-type systems [7,14]. The results demonstrate that SCOBY fermentation substantially altered the chemical composition of both red and white gooseberry beverages, while the magnitude and pattern of these changes depended on the juice matrix and carbon source. These compositional changes provide the chemical context for the fermentation-induced modifications in phenolic profiles described in the following section.

### 2.3. Transformation of Polyphenolic Compounds

SCOBY fermentation markedly modified the polyphenolic profile of gooseberry-based beverages, although the direction and magnitude of these changes varied among individual compounds and formulations (Table 3 and Supplementary Table S1).

The total concentration of identified polyphenolic compounds increased during fermentation in all variants, from 777.24–1220.89 at D_0 to 980.92–1859.69 at D_10. The increase was more pronounced in red gooseberry juice (RGJ), particularly in the honey-supplemented variant, which showed the highest total concentration at the end of fermentation.

Phenolic acids and their derivatives exhibited compound-specific changes rather than a uniform response to fermentation. Several abundant caffeoyl derivatives, including caffeoyl malate and caffeoylquinic acids, progressively decreased, whereas other compounds, such as quinic acid derivatives, trigalloyl quinic acid, selected syringic and ferulic acid derivatives, and 3,4-dimethoxycinnamic acid derivatives, increased during fermentation. These opposing trends indicate extensive rearrangement of the phenolic acid profile rather than a general increase in this compound class. Such changes may reflect microbial hydrolysis and transformation of conjugated phenolics and structural modifications within the plant matrix [2,6,15]. Differences between sucrose- and honey-supplemented variants may additionally reflect differences in matrix composition and fermentation metabolism [16].

The most pronounced fermentation-induced changes were observed among flavan-3-ols and related compounds. Between D_0 and D_10, (−)-epigallocatechin increased 1.80-fold in WGJ-S (from 68.09 to 122.47 mg/L) and 2.23-fold in RGJ-H (from 184.74 to 411.58 mg/L). An even greater increase was observed for (−)-epigallocatechin gallate, which increased 3.58-fold in WGJ-S (from 72.70 to 260.24 mg/L) and 7.20-fold in RGJ-H (from 61.78 to 444.59 mg/L). The compound tentatively identified as gallocatechin gallate (GCG; unknown isomer) increased in all formulations, with fold changes of 2.09 and 2.28 in WGJ-S and WGJ-H, respectively, and substantially greater increases of 8.67 and 9.13 in RGJ-S and RGJ-H, respectively. In contrast, the B-type procyanidin dimer and procyanidin tetramer generally decreased during fermentation, particularly in RGJ-based beverages. These opposing trends suggest a redistribution of flavan-3-ol-related compounds during fermentation, potentially associated with the release of phenolics from the fruit matrix and microbial or enzymatic transformations of condensed phenolic structures [2,6]. However, the specific pathways responsible for these changes could not be established from the present data.

Anthocyanins were detected exclusively in RGJ-based beverages and were comparatively stable during fermentation. However, their response was compound-dependent. Peonidin-3-glucoside decreased slightly, whereas cyanidin-3-xyloside, peonidin-3-rutinoside, cyanidin glycoside, and cyanidin-coumaroyl-glucopyranoside remained relatively stable or increased by D_10. Thus, the applied fermentation conditions did not result in an overall depletion of the detectable anthocyanin fraction. Interactions with other phenolic constituents, including flavan-3-ols, may contribute to anthocyanin stability in fermented matrices [17].

Flavonols showed a more heterogeneous response than suggested by their overall class designation. Several abundant glycosides, including quercetin 3-*O*-rutinoside, isorhamnetin 3-*O*-rutinoside, quercetin-3-*O*-rhamnoside, and isorhamnetin 3,7-di-*O*-hexoside, decreased during fermentation. In contrast, quercetin 7-*O*-glucoside, quercetin 3-*O*-neohesperidoside, taxifolin 3-*O*-glucoside, and, particularly in RGJ, quercetin-3-O-sambubioside increased. These compound-specific changes are consistent with the occurrence of simultaneous degradation and conversion processes within the flavonoid fraction, potentially involving microbial transformation of glycosylated phenolics [2,18].

Theaflavin-related compounds were detected at comparatively low concentrations. Theaflavin-3,3′-gallate generally increased during fermentation, whereas the response of the unidentified theaflavin isomer was less consistent. Because black-tea kombucha was used as the liquid starter, the presence of these tea-associated compounds may at least partly reflect their introduction with the starter rather than their de novo formation in the gooseberry matrix [16].

An important consideration in interpreting the phenolic profiles is the use of previously fermented black tea kombucha as the liquid starter. This starter may have contributed tea-associated phenolic compounds, including epigallocatechin derivatives and theaflavins, to the gooseberry-based beverages. Therefore, the detection of these compounds cannot be attributed exclusively to the gooseberry juice matrix or to their formation during fermentation. Although the same batch and concentration of starter were used in all experimental formulations, providing a consistent starting contribution across treatments, the starter itself was not subjected to separate phytochemical characterization. Moreover, no starter-specific experimental control was included. Consequently, the relative contributions of the gooseberry matrix, starter-derived compounds, and fermentation-associated transformations to the observed phenolic profiles cannot be fully distinguished. These considerations are particularly relevant when interpreting the biological activities of the fermented beverages.

Overall, SCOBY fermentation caused a substantial redistribution of the polyphenolic profile rather than a uniform increase across all phenolic classes. The increase in total identified compounds was accompanied by pronounced accumulation of several flavan-3-ol-related compounds, decreases in oligomeric procyanidins and selected phenolic acid and flavonol derivatives, and relative preservation of the anthocyanin fraction in RGJ-based beverages. These compositional changes provide a basis for examining whether fermentation-induced modifications of the phenolic profile were accompanied by changes in the biological activities of the beverages.

### 2.4. Volatile Compounds Profile

The volatile compound profile of gooseberry-based beverages differed markedly among juice and carbon-source combinations and showed formulation-dependent changes during SCOBY fermentation (Table 4; Supplementary Tables S2 and S3). The identified volatile organic compounds (VOCs) comprised mainly alcohols and esters, together with acids, aldehydes, ketones, terpenes and terpenoids, and furan derivatives. Esters and alcohols are important contributors to the aroma profile of kombucha and other fermented beverages [19].

It should be emphasized that the alcohols reported in Table 4 represent individual volatile alcohols identified by HS-SPME Arrow-GC–MS and do not include ethanol, which was determined separately using a different analytical technique (Table 2). Therefore, changes in the concentrations of these volatile alcohols should not be directly compared with ethanol accumulation during fermentation. Furthermore, D_0 samples were collected after the addition of the liquid kombucha starter, which may have contributed to the initial volatile profile alongside compounds originating from gooseberry juice and the added sweetener. The relatively high concentrations of certain volatile alcohols at D_0 and their limited changes during fermentation are therefore not necessarily inconsistent with the substantial increase in ethanol concentration observed in Table 2.

RGJ-based beverages were characterized by a comparatively high contribution of esters to the volatile profile. In RGJ-S, total ester concentration increased during the first five days of fermentation and remained at a similar level at D_10. This change was largely associated with increases in several ethyl esters, including ethyl acetate, ethyl octanoate, ethyl decanoate, and ethyl dodecanoate. In contrast, the ester fraction of RGJ-H remained comparatively stable throughout fermentation, demonstrating that changes in the volatile profile depended not only on the fruit matrix but also on the added carbon source. Several of these esters are associated with fruity aroma characteristics in fermented beverages [19]. Their occurrence and changes during fermentation are consistent with the formation and transformation of volatile esters associated with microbial metabolism.

WGJ-based beverages showed a distinctly different volatile profile, with alcohols representing the major VOC fraction. However, the effect of fermentation strongly depended on the carbon source. In WGJ-S, the total VOC concentration decreased markedly between D_0 and D_5 and subsequently remained relatively stable until D_10. This decrease involved several volatile classes, including alcohols, esters, acids, aldehydes, and terpenes and terpenoids. In contrast, the overall volatile profile of WGJ-H changed comparatively little during fermentation, and alcohols remained the predominant group throughout the process. The detailed profiles included higher alcohols and other alcohol-derived volatiles that may contribute to the characteristic aroma of WGJ-based beverages (Supplementary Table S2). Generally, SCOBY fermentation did not produce a uniform increase in volatile compounds but rather induced formulation-dependent remodeling of the VOC profile. The clearest fermentation-associated changes were observed in WGJ-S, where the total concentration of detected volatiles decreased, and in RGJ-S, where the ester fraction increased. By contrast, the honey-supplemented variants showed comparatively smaller changes in total VOC abundance.

These differences are consistent with the dependence of volatile formation on substrate composition and microbial metabolism during fermentation [20,21]. The resulting differences in VOC composition may contribute to differences in aroma perception among the beverages, which are considered together with the sensory results below.

The relatively limited changes in the concentrations of several volatile compound groups, particularly in honey-supplemented beverages, should not be interpreted as evidence of metabolic inactivity. The evolution of the volatile profile was formulation-dependent, with an increase in the ester fraction in RGJ-S and a marked decrease in total detected VOCs in WGJ-S, whereas the honey-supplemented variants exhibited comparatively stable profiles. Importantly, the measured concentration of an individual volatile compound reflects its net accumulation and does not directly indicate its rates of formation and consumption. Consequently, relatively stable concentrations may be compatible with simultaneous production and transformation processes, although these mechanisms were not directly investigated in the present study. Furthermore, the pronounced changes in sugar utilization, ethanol accumulation, and organic acid concentrations demonstrate that substantial biochemical transformations occurred without necessarily producing proportional changes in all monitored volatile compounds. The limited temporal variation observed for some VOC groups therefore represents a characteristic of the measured profiles rather than evidence of an absence of fermentation. Nevertheless, the underlying metabolic mechanisms cannot be conclusively established from the available data.

### 2.5. Antioxidant and Enzyme Inhibitory Activities

SCOBY fermentation was associated with increased antioxidant capacity and enzyme inhibitory activities of gooseberry-based beverages, although the magnitude of these changes differed among juice and carbon-source combinations (Table 5). Antioxidant capacity measured by DPPH, ABTS, and FRAP generally increased during fermentation, with the highest values observed at D_10. RGJ-based beverages consistently exhibited higher antioxidant capacity than the corresponding WGJ variants, while honey-supplemented beverages generally showed higher values than their sucrose-supplemented counterparts. The strongest antioxidant response was observed in RGJ-H, in which DPPH, ABTS, and FRAP values increased from 145.26, 202.35, and 320.51 µmol Trolox/100 mL at D_0 to 165.37, 251.36, and 451.22 µmol Trolox/100 mL at D_10, respectively. Similar increases in antioxidant activity have been reported for fermented fruit matrices and kombucha-type beverages and have been associated with fermentation-induced transformations and increased availability of low-molecular-weight phenolic compounds [3,7].

Fermentation was also accompanied by increased inhibition of cyclooxygenase enzymes. COX-1 inhibition increased in all formulations and reached the highest value in RGJ-H at D_10 (92.99%), while COX-2 inhibition showed a more moderate increase, reaching 52.12% in the same beverage. RGJ-based samples generally exhibited greater COX inhibitory activity than WGJ-based beverages, and the highest values were consistently observed in the honey-supplemented RGJ variant. These results indicate increased in vitro cyclooxygenase-inhibitory potential following fermentation. The particularly high activity of RGJ-H may reflect the combined contribution of its fermentation-modified phytochemical constituents; however, the present assays do not allow the effects to be attributed to individual compounds or to establish synergistic interactions [22].

In undiluted samples, inhibition of cholinesterases also increased during fermentation. AChE inhibition increased from 9.28–22.89% at D_0 to 11.90–35.18% at D_10, whereas BuChE inhibition increased from 11.89–29.56% to 18.00–36.19%. Again, RGJ-H exhibited the highest inhibitory activity at the end of fermentation. In contrast, inhibition of carbohydrate-hydrolyzing enzymes by the undiluted samples remained comparatively low. α-Amylase inhibition ranged from 2.05–5.54% at D_0 and increased to 3.04–6.25% at D_10, whereas α-glucosidase inhibition increased more modestly, from 7.18–11.06% to 7.88–12.08%. Thus, although fermentation enhanced the measured inhibitory activity toward both enzymes, the magnitude of these effects was substantially lower than that observed for COX-1 or the cholinesterases. Given the low inhibition percentages observed even in undiluted beverage samples, these findings should be interpreted as limited in vitro enzyme-inhibitory activity and do not provide sufficient evidence to support claims of meaningful antidiabetic efficacy or glucose-lowering effects in vivo.

The differences in the magnitude of the observed functional responses may also reflect the distinct principles underlying the applied assays. DPPH, ABTS, and FRAP primarily assess chemical antioxidant properties, whereas enzyme inhibition depends on interactions between beverage constituents and specific enzyme targets under the assay conditions. Consequently, fermentation-induced changes in the abundance and composition of phenolic compounds would not necessarily produce proportional changes across these assays. In particular, the relatively weak inhibition of α-amylase and α-glucosidase, despite increased antioxidant capacity, highlights the importance of distinguishing chemical antioxidant responses from enzyme-specific inhibitory effects. The present findings therefore support a formulation-dependent functional profile rather than a uniform enhancement of all measured biological activities.

In summary, fermentation-induced changes in biological activity were strongly dependent on the beverage formulation. RGJ-based beverages, particularly RGJ-H, showed the highest antioxidant capacity and enzyme inhibitory activities at D_10. These functional changes coincided with the extensive remodeling of the phenolic profile observed during fermentation, particularly the accumulation of several flavan-3-ol-related compounds. However, the present data demonstrate an association between compositional and functional changes rather than a direct causal relationship.

### 2.6. Effects of Polyphenolic Extracts on HepG2 Cells

#### 2.6.1. Cell Growth and Viability

**Figure 1:**
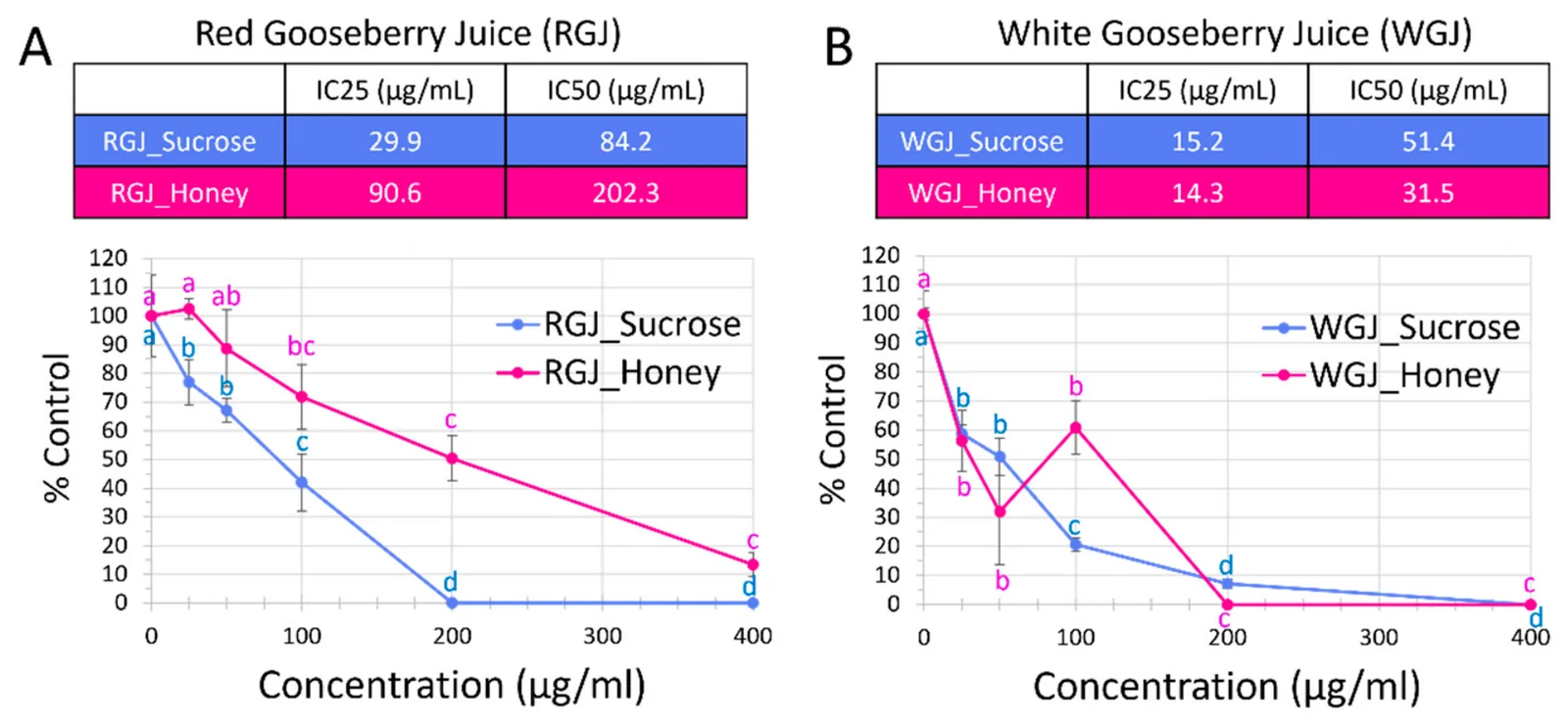
Growth inhibition of HepG2 liver cancer cells treated with polyphenolic extracts from fermented gooseberry juices: (**A**) red gooseberry juice (RGJ) fermented with sucrose (RGJ_S) or honey (RGJ_H); (**B**) white gooseberry juice (WGJ) fermented with sucrose (WGJ_S) or honey (WGJ_H). Viable and dead cells were quantified using the trypan blue exclusion assay. The IC25 and IC50 values represent extract concentrations (µg/mL) causing 25% and 50% reductions in cell growth, respectively, while maintaining at least 90% cell viability. Results are expressed as mean ± SD from three independent experiments. Different letters (a–d) indicate statistically significant differences between groups, as determined by one-way ANOVA followed by Tukey’s post hoc test (*p* < 0.05).

To further evaluate whether the observed differences in chemical composition and enzyme inhibitory activities translate into cellular effects, the impact of fermented gooseberry extracts on HepG2 cell growth and viability was assessed. Unlike the antioxidant and enzyme inhibition assays performed directly on the beverages, the cellular experiments were conducted using isolated polyphenolic extracts; therefore, the observed cellular responses should be interpreted specifically in relation to the extractable phenolic fraction rather than the whole fermented beverage. We aimed to compare the effects of the phenolic-enriched extracts isolated from kombucha-fermented red and white gooseberry juices on HepG2 viability and growth. Lower values for IC25 and IC50 concentrations corresponded to a more potent effect. When comparing the juice samples (Figure 1A,B), WGJ was found to have a stronger effect on HepG2 cell growth compared to the RGJ samples, with the WGJ with honey having the lowest IC25 of 14.3 µg/mL and WGJ with sucrose having an IC25 of 15.2 µg/mL. The corresponding IC50 values (31.5 and 51.4 µg/mL, respectively) were also lower than those determined for the RGJ-derived extracts (202.3 and 84.2 µg/mL for RGJ with honey and sucrose, respectively), further demonstrating the greater growth-inhibitory potency of the WGJ-derived extracts under the applied experimental conditions. For this reason, cells treated with the WGJ samples were selected for later gene expression experimentation.

Type of sweetener used as a fermentation substrate did not appear to play a major role in the effects of the extracts on cell growth, with no pattern in either a strengthening or weakening of potency direction being observed. Both sucrose- and honey-sweetened samples were selected for the next phase of gene expression experimentation. Most samples displayed little-to-no cells at 200–400 µg/mL (Figure 1), illustrating a possible cytotoxic effect of kombucha polyphenol extracts at high concentrations. Because cell growth inhibition at the highest extract concentrations may reflect cytotoxicity rather than a selective antiproliferative response, subsequent experiments were conducted at a substantially lower concentration.

Therefore, we have selected 25 µg/mL dose for further experiments. For cell growth, we found that polyphenolic extracts made from WGJ fermentation products (Figure 1B) had more potent effects on slowing and disrupting HepG2 cell growth compared to polyphenolic extracts made from RGJ (Figure 1A). This observation contrasts with the trends observed in antioxidant capacity and total polyphenolic content, where RGJ samples consistently exhibited higher values, indicating that cellular responses are not directly proportional to bulk compositional parameters. This divergence is particularly relevant because it indicates that total phenolic concentration and chemical antioxidant capacity alone are insufficient predictors of the cellular response to the polyphenolic extracts.

This result appears to be in opposition to findings that RGJs have both higher antioxidant capacity and more polyphenolic compounds than WGJs, including resveratrol which has been shown to improve glucose metabolism and lipid profile, and reduce liver fibrosis and steatosis in previous studies [23]. However, polyphenolic profile is likely altered during fermentation and by interactions with the other compounds in kombucha. During kombucha production, microbial fermentation can transform polyphenolic compounds into new derivatives with altered biological properties [3].

Additionally, interactions between polyphenols and other fermentation by-products—such as organic acids, ethanol and microbial metabolites—may influence their cellular effects. However, because the HepG2 experiments were performed with polyphenolic extracts rather than whole beverages, the direct contribution of ethanol, organic acids, and other non-extracted fermentation metabolites to the observed cellular effects cannot be established from the present experiment. These effects highlight the importance of fermentation-driven compositional complexity and potential synergistic interactions within the matrix. Indeed, our current analysis shows that WGJ fermentation products contain around 50% and 40% less flavan-3-ols and total phenolics, respectively, and have undetectable levels of anthocyanins, as compared to RGJ (Table 3). However, the content of phenolic acids was comparable between white and red gooseberry juice samples. Although WGJ samples had on average lower antioxidant, anti-inflammatory, neuroprotective and anti-diabetic activities compared with RGJ, the values were still substantial (Table 4) and not statistically different from those for RGJ (*p* > 0.05).

The potency of WGJ-derived extracts suggests that the fermentation of WGJ may lead to formation of derivatives of bioactive compounds and their matrix that would be particularly effective at disrupting cellular growth of liver cells. Accordingly, the stronger effect of WGJ-derived extracts should not be interpreted as resulting from a greater overall abundance of phenolics, but may instead reflect differences in the qualitative composition of the extracted phenolic fraction and/or interactions among its constituents. Future research should incorporate non-targeted full polyphenolic profiling of each kombucha extract in search of new derivatives that could explain observed distinct biological effects.

#### 2.6.2. Gene Expression

To further elucidate the molecular mechanisms underlying the observed antiproliferative effects, gene expression analysis was performed. Because these experiments were performed only with WGJ-derived extracts at a single selected concentration, the gene-expression results provide mechanistic context for the WGJ treatments rather than a direct comparison between red and white gooseberry matrices. In our current study, phenolic extracts from gooseberry juice fermented beverages were characterized by antioxidant, anti-inflammatory, neuroprotective and anti-diabetic activities, along with the inhibition of growth of cancer cells. To provide a molecular explanation for the observed potency, we tested the effect of the extracts on expression of a set of genes in HepG2 liver cancer cells as a model of disrupted liver homeostasis. We proceeded with samples that were the most potent in inhibition of cell growth (the lowest IC50 value), namely 100% WGJ with sucrose or honey. We selected *G6PC*, *FABP4*, *ALDH1L1*, and *SERPINE1* genes based on their differential expression in RNA sequencing data following pterostilbene treatment, a dietary polyphenol, in an in vivo rat model of liver cancer in our previous study [24]. *WNT10B* and *EPCAM* were included as liver-relevant members of the Wnt signaling [25,26], and because the Wnt pathway was a major oncogenic pathway inhibited by polyphenols in our previous work [27]. An additional criterion was the involvement of the genes in regulation of metabolism, signaling molecules and/or oncogenic signaling pathways, which are linked to the tested biological activities and are specifically crucial for normal liver functions.

**Figure 2:**
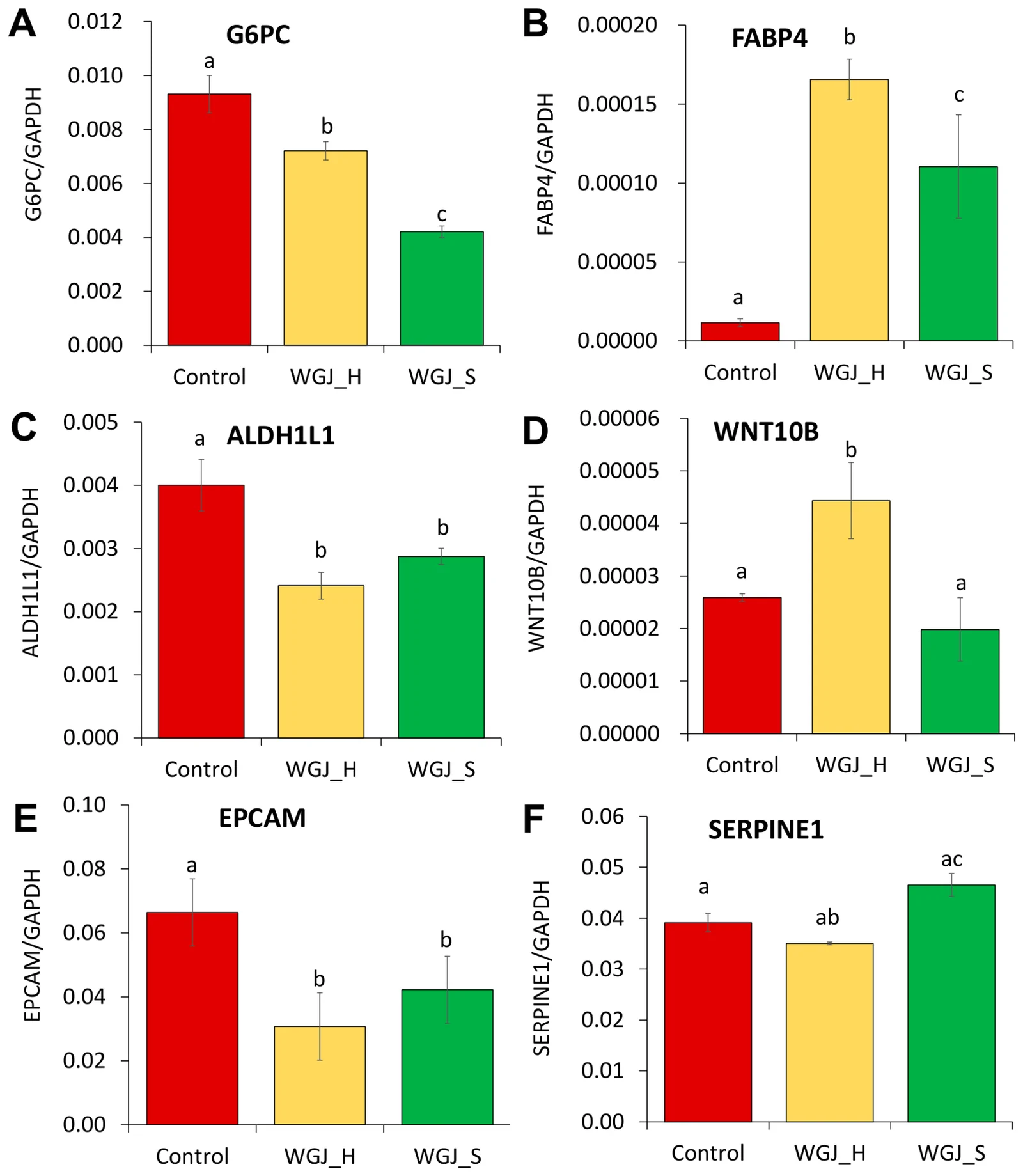
Effects of polyphenolic extracts from fermented white gooseberry juice (WGJ) on the expression of genes involved in metabolic regulation and oncogenic signaling in HepG2 liver cancer cells: (**A**) G6PC; (**B**) FABP4; (**C**) ALDH1L1; (**D**) WNT10B; (**E**) EPCAM; and (**F**) SERPINE1. HepG2 cells were treated for 4 days with 25 µg/mL of polyphenolic extracts obtained from white gooseberry juice fermented with honey (WGJ_H) or sucrose (WGJ_S). Gene expression was determined by quantitative reverse transcription PCR (qRT-PCR) and normalized to *GAPDH* expression. Results are expressed as mean ± SD from three independent experiments. Different letters (a–c) indicate statistically significant differences between groups, as determined by one-way ANOVA followed by Tukey’s post hoc test (*p* < 0.05).

Treating cells with extracts from WGJ fermented with honey or sucrose led to a 30–50% downregulation of *G6PC* (Figure 2A), a gene coding for glucose-6-phosphatase that is involved in glucose metabolism and lipid homeostasis [28]. Treatment with WGJ led to more than 10-fold increase in expression for *FABP4* (Figure 2B), a gene linked to increased lipid droplet accumulation in the liver as is seen in metabolic dysfunction-associated steatotic liver disease [29] that has also been found to be elevated in cases of hepatocellular carcinoma [30]. Thus, the transcriptional response was not uniformly directed toward changes that could be interpreted as beneficial, emphasizing the complexity of the cellular response to these extracts. In the analysis of *ALDH1L1* (Figure 2C), a gene involved in tumour suppression [31], folate metabolism and epigenetic equilibrium [32], both WGJ sweetened with sucrose and WGJ with honey led to significant downregulation of the gene.

Expression of *WNT10B* (Figure 2D), an oncogene from the Wnt signaling pathway [25], was significantly upregulated by treatment with WGJ with honey, resulting in 55% increased *WNT10B* expression. Treatment with extracts from WGJ sweetened with sucrose did not result in statistically significant changes in expression of *WNT10B*, compared to control. Interestingly, *EPCAM*, a gene with an oncogenic potential that is positively regulated by Wnt signaling and elevated in liver cancer [26], was found to be downregulated by approximately 50% in both treatments compared to the control (Figure 2E). The opposite responses of *WNT10B* and *EPCAM*, particularly following treatment with the honey-derived extract, indicate that the effects cannot be interpreted as uniform suppression of WNT-related signaling.

*SERPINE1* is a gene that codes for plasminogen activator inhibitor 1 (PAI-1) that is linked to decreased hepatic lipid metabolism and aberrant mammalian lipid homeostasis [33]. While PAI-1 plasma levels are high in experimental models of obesity and diabetes, mice deficient in PAI-1 are resistant to diet-induced obesity and hepatic steatosis [33]. There was no difference in *SERPINE1* expression between the control and the samples treated with WGJ with sucrose or honey, although a slight non-statistically significant decrease was detected for WGJ fermented with honey (Figure 2F). Importantly, the level of *SERPINE1* remained significantly lower after WGJ with honey as compared with WGJ with sucrose.

Polyphenolic extracts derived from 100% WGJ were found to modulate expression of the selected genes (Figure 2), which may partially explain their inhibitory effects on the growth of HepG2 cells (Figure 1). However, the direction of these transcriptional changes differed among the analyzed genes, indicating a complex rather than uniformly inhibitory molecular response. Given the roles of *EPCAM* and *SERPINE1*, their downregulation could contribute to the suppression of pro-migratory and pro-diabetic signaling pathways. Notably, extracts obtained from fermentations with both honey and sucrose resulted in approximately 50% downregulation of *EPCAM* (Figure 2E), a downstream target of Wnt signaling. This reduction may indicate inhibition of the Wnt pathway; however, *WNT10B* expression was not suppressed by any of the extracts (Figure 2D). Accordingly, the present gene-expression data do not provide evidence for generalized inhibition of the WNT pathway and should instead be interpreted at the level of the individual genes examined. Although *WNT10B* is frequently overexpressed in liver cancer and associated with tumorigenesis, it has also been reported to exhibit a bifunctional role, including the ability to reduce cell proliferation via Wnt-independent mechanisms [34]. In addition, *G6PC* and *ALDH1L1*, which are involved in metabolic homeostasis, were both downregulated following treatment with the extracts (Figure 2A,C). Inhibition of *G6PC* by polyphenols has previously been linked to improved insulin sensitivity. Specifically, polyphenol-rich extracts have been shown to decrease *G6PC* activity, thereby reducing hepatic glucose output and enhancing glycogen storage [35]. Downregulation of *ALDH1L1*, in turn, may result from the ability of polyphenols to act as inhibitors of NADP(+) that is essential for *ALDH1L1* activity [36].

An unexpected result noted in our study was WGJ polyphenolic extract-mediated upregulation of *FABP4* (Figure 2B), a gene functionally linked to steatosis and demonstrated to be inhibited in the liver by numerous polyphenols [37]. Nevertheless, increased levels of *FABP4* along with simultaneously observed inhibition of cell growth could indicate the activation of signaling towards fatty acid transport in hepatocytes which is the primary function of *FABP4* [38,39]. In addition, the upregulation of *FABP4* could be a short-term effect of polyphenols as a result of their pleiotropic action and possible feedback from other affected pathways, and may lessen with long-term exposure. Nevertheless, the present experiment does not establish whether the observed *FABP4* response is transient, and this interpretation would require time-course experiments.

General, the gene expression results support the hypothesis that fermentation-derived transformations, rather than total polyphenol content alone, play a key role in determining the biological activity of gooseberry-derived kombucha extracts. The observed modulation of metabolic and oncogenic pathways further highlights the potential of these fermented products as sources of bioactive compounds with complex cellular effects. Taken together, the cell-growth and gene-expression results indicate that the biological potency of the extracts was not predicted by total phenolic content or antioxidant capacity alone. At the same time, the heterogeneous transcriptional responses observed across the selected genes warrant cautious interpretation and do not support a uniformly beneficial or anticancer molecular effect.

### 2.7. Sensory Analysis of Fermented Gooseberry Beverages

**Figure 3:**
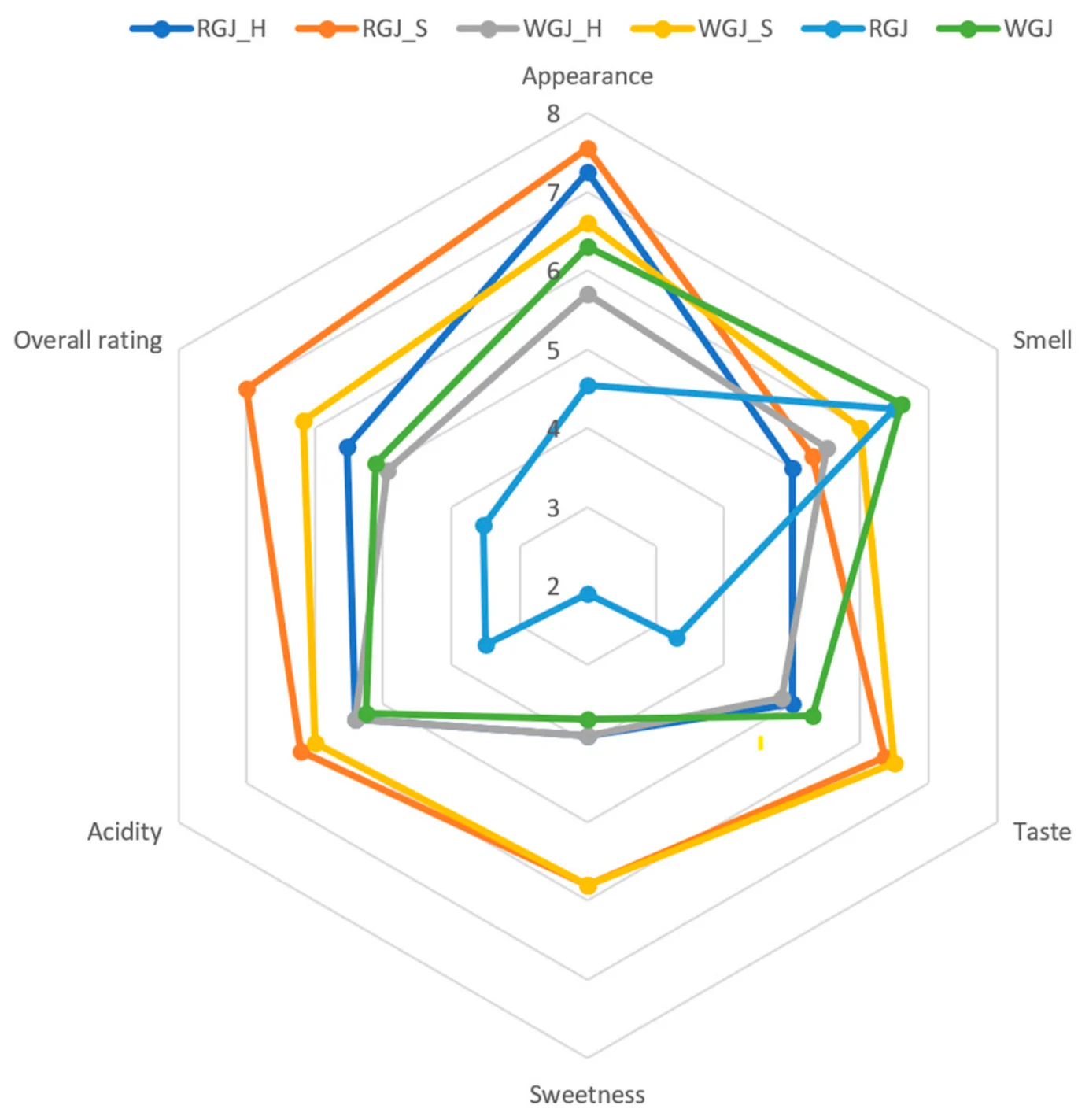
Sensory evaluation of fermented gooseberry beverages and unfermented gooseberry juices. Sensory attributes, including appearance, smell, taste, sweetness, acidity, and overall acceptability, were evaluated using a 9-point hedonic scale, where 1 indicates “dislike extremely” and 9 indicates “like extremely”. Higher scores indicate greater sensory acceptance. Abbreviations: WGJ_S – white gooseberry juice fermented with sucrose; WGJ_H – white gooseberry juice fermented with honey; RGJ_S – red gooseberry juice fermented with sucrose; RGJ_H – red gooseberry juice fermented with honey; RGJ – unfermented red gooseberry juice; WGJ – unfermented white gooseberry juice.

Sensory evaluation revealed distinct acceptance profiles among the six gooseberry-based beverages (Figure 3). The effect of fermentation and formulation was particularly pronounced for red gooseberry juice. Unfermented RGJ received the lowest scores for most evaluated attributes, whereas both fermented RGJ formulations showed substantially more favorable sensory profiles. Among all beverages, RGJ-S received the highest scores for appearance and overall rating and was also highly rated for taste and acidity. WGJ-S showed a similarly favorable profile, particularly for taste and sweetness.

The influence of the carbon source differed depending on the sensory attribute and juice matrix. Sucrose-supplemented fermented beverages received higher scores for taste, sweetness, acidity, and overall rating than the corresponding honey-supplemented formulations. In contrast, differences in smell followed a different pattern, and unfermented WGJ was among the most favorably rated samples for this attribute. Thus, the sensory response cannot be described as a uniform improvement across all attributes following fermentation, but rather as an attribute- and matrix-dependent change in consumer acceptance.

The sensory differences among the beverages coincided with substantial differences in their chemical and volatile profiles. RGJ-based fermented beverages were characterized by a greater contribution of esters to the volatile fraction, including several compounds associated with fruity aroma characteristics, whereas alcohols represented a larger proportion of the WGJ volatile profile. However, the relatively high smell rating of unfermented WGJ demonstrates that the overall hedonic perception of aroma cannot be directly predicted from the abundance of individual volatile classes. The observed sensory response likely reflects the combined contribution of multiple volatile and non-volatile constituents and their interactions. The interplay between organic acids and volatile compounds has previously been reported to play an important role in shaping flavor perception in fermented beverages [40].

The sensory acceptance results require particular caution when considered alongside the physicochemical characteristics of the fermented beverages. After 10 days of fermentation, the beverages exhibited substantial sugar depletion, low pH values (approximately 2.7–2.8), and ethanol concentrations ranging from 4.27 to 6.28% (Table 2). These characteristics would generally be expected to influence taste perception and may limit consumer acceptance. Nevertheless, some fermented formulations received relatively favorable hedonic ratings for sweetness, acidity, and overall acceptability within the investigated panel. Although the nine-point hedonic scale measured liking rather than perceived intensity, this distinction alone does not explain the apparent discrepancy between the chemical composition and the sensory ratings. The present study did not include descriptive sensory profiling, intensity measurements, or instrumental–sensory correlation analyses that would allow the underlying reasons for these responses to be identified. Furthermore, the evaluation involved only 20 untrained consumers, and individual preferences or differences in sensory sensitivity may have influenced the results. Therefore, the sensory findings should be regarded as preliminary observations specific to the investigated panel and experimental conditions. They should not be interpreted as evidence that the beverages possessed high perceived sweetness, low perceived acidity, or broad consumer acceptability. Further sensory studies involving a larger and more diverse panel, together with descriptive analysis of sweetness, sourness, and ethanol-related sensations, are needed to clarify these findings.

Notably, the formulation showing the highest functional properties was not identical to that showing the most favorable sensory profile. RGJ-H exhibited particularly high antioxidant capacity and enzyme inhibitory activities, whereas RGJ-S received the highest overall sensory rating. This divergence indicates a potential trade-off between fermentation-derived functional characteristics and consumer acceptance and highlights the importance of considering both aspects when developing fruit-based fermented beverages.

Overall, the sensory results demonstrate that the response to fermentation was dependent on both the gooseberry matrix and the sensory attribute evaluated. The most pronounced improvement was observed for RGJ, particularly when sucrose was used as the added carbon source, whereas WGJ showed a more attribute-specific response. Among the tested formulations, RGJ-S exhibited the most favorable overall sensory profile.

A comparison of the four fermented formulations revealed that the effects of SCOBY fermentation depended on both gooseberry juice type and carbon source, with different formulations performing more favorably depending on the evaluated endpoint. Red gooseberry beverages generally exhibited higher concentrations of identified phenolic compounds and greater antioxidant capacity than their white gooseberry counterparts, with the honey-supplemented red gooseberry formulation (RGJ-H) showing the highest final phenolic concentration and particularly strong antioxidant responses. However, these compositional advantages did not translate into the strongest inhibition of HepG2 cell growth. Instead, polyphenolic extracts obtained from fermented white gooseberry beverages, particularly WGJ-H and WGJ-S, exhibited greater growth-inhibitory activity in this cell model. Sensory acceptance followed a different pattern, with the sucrose-supplemented red gooseberry formulation (RGJ-S) receiving the highest overall rating and WGJ-S also showing favorable acceptance of taste and sweetness. Collectively, these findings indicate that the most favorable formulation depends on the intended product characteristic and that phenolic abundance, antioxidant capacity, cellular responses, and sensory acceptance should be considered as distinct outcomes rather than interchangeable indicators of beverage quality.

## 3. Materials and Methods

### 3.1. Materials

Commercial red and white gooseberry (*Ribes uva-crispa*) juices were obtained from Jóźwiak Sady Katarzyna Jóźwiak (Mogielnica, Poland). The juices were stored at 4 °C and used within their declared shelf life. Food-grade sucrose and buckwheat honey, purchased from a local retail market, were used as additional carbon sources for fermentation.

A commercially available kombucha symbiotic culture of bacteria and yeast (SCOBY) was used as the fermentation starter according to the manufacturer’s instructions. According to the manufacturer’s specification, the SCOBY contained the yeasts *Saccharomyces bisporus*, *Saccharomyces cerevisiae*, *Saccharomycodes ludwigii*, *Torulopsis* sp., and *Zygosaccharomyces* sp., as well as acetic acid bacteria belonging to the genus *Acetobacter*. The symbiotic culture of bacteria and yeast (SCOBY) used for kombucha fermentation was obtained from a commercial supplier. Prior to fermentation, the SCOBY was visually inspected for signs of contamination.

### 3.2. Fermentation Process

Fermentation substrates were prepared by supplementing red and white gooseberry juices with either sucrose (80 g/L) or buckwheat honey (80 g/L). The substrates were thoroughly mixed until the added carbon source was completely dissolved. Previously fermented black tea kombucha was used as the liquid starter at a concentration of 100 mL/L, followed by inoculation with 100 g of SCOBY. The same batch of liquid starter was added at an identical concentration to all four experimental formulations (RGJ-S, RGJ-H, WGJ-S, and WGJ-H). In addition to introducing fermentation-associated microorganisms and metabolites, the black tea-based starter represented a potential source of tea-derived phenolic compounds in the resulting beverages. Each fermentation was conducted at a working volume of 1 L in a sterile glass vessel covered with sterile gauze to allow air exchange while minimizing external contamination. The fermentation mixtures were incubated under static conditions at 23 °C for 10 days.

Samples were collected at three time points: immediately after preparation of the fermentation mixture, including the addition of the carbon source and liquid starter, but before the onset of fermentation (D_0), and after 5 (D_5) and 10 (D_10) days of fermentation. Samples collected at all three time points were subjected to physicochemical, phytochemical, and in vitro functional analyses. Sensory evaluation included the unfermented red and white gooseberry juices as reference samples and the four beverages collected after 10 days of fermentation. Three independent fermentations were performed for each combination of gooseberry juice type and carbon source, with each 1-L fermentation representing an independent biological replicate.

### 3.3. Microbiological Analysis

Microbiological enumeration was performed immediately after the initiation of fermentation and after 10 days of incubation at 23 °C. Homogenized samples were serially diluted and plated in triplicate for each culture medium. Total viable counts (TVC) were determined using Plate Count Agar under aerobic conditions at 30 °C for 48 h. Presumptive acetic acid bacteria (AAB) were enumerated on GYC agar containing glucose, yeast extract, and CaCO_3_, supplemented with acetic acid and an antifungal agent to enhance selectivity, following aerobic incubation at 30 °C for up to 5 days. Presumptive lactic acid bacteria (LAB) were enumerated on MRS agar (pH approximately 5.5) under microaerophilic/anaerobic conditions at 30 °C for 48–72 h. Yeasts and filamentous fungi were enumerated on YGC agar under aerobic conditions at 25–28 °C for 3–5 days. Appropriate sterility controls were included for each batch of analyses. Microbial counts were expressed as colony-forming units per milliliter (CFU/mL).

### 3.4. Basic Chemical Composition

The basic chemical composition of the samples was characterized by determining pH, organic acid and sugar profiles, and ethanol concentration. The pH was measured at room temperature using a TitroLine 6000 pH meter (SI Analytics GmbH, Mainz, Germany) after sample homogenization. Organic acids were analyzed by high-performance liquid chromatography (HPLC) according to the method described by Chandran et al. [41]. Separation was performed using an Aminex HPX-87H column (Bio-Rad, Hercules, CA, USA) maintained at 65 °C, with 0.001 N H_2_SO_4_ as the mobile phase. Sugars were determined according to Kolniak-Ostek [42] using a Merck-Hitachi HPLC system equipped with an evaporative light-scattering detector (ELSD). Quantification was performed using calibration curves prepared for glucose, fructose, sorbitol, and sucrose (R^2^ = 0.9999). Ethanol concentration was determined by near-infrared spectroscopy using an Anton Paar DMA 4500 instrument (Anton Paar, Graz, Austria), according to Gasiński et al. [43]. Prior to analysis, the samples were degassed, filtered through laboratory filter paper, and equilibrated to 20 °C. For each independent fermentation, analytical measurements were performed in triplicate.

### 3.5. UPLC-PDA-Q/ToF-MS Analysis of Phenolic Compounds

Phenolic compounds were analyzed using an ACQUITY UPLC system coupled with a photodiode array (PDA) detector and a quadrupole time-of-flight mass spectrometer (Q-ToF-MS) (Waters, Manchester, UK), according to the method described by Chandran et al. (2025) [41]. Prior to analysis, the samples were centrifuged and filtered through a 0.22 µm membrane filter. Chromatographic separation was performed using a UPLC BEH C18 column (1.7 µm, 2.1 × 100 mm; Waters) maintained at 30 °C. The mobile phase consisted of 0.1% (*v*/*v*) formic acid (solvent A) and acetonitrile (solvent B), with a flow rate of 0.42 mL/min. Mass spectrometric detection was performed using electrospray ionization (ESI) in negative-ion mode over an *m*/*z* range of 100–1500.

Data were acquired and processed using MassLynx 4.0 and ChromaLynx 4.0 software (Waters, Manchester, UK). Phenolic compounds were identified based on retention times, UV–Vis spectral characteristics, accurate mass measurements, MS/MS fragmentation patterns, and comparison with authentic standards, when available, and literature data. PDA monitoring was performed at 280 nm for flavan-3-ols, 320 nm for phenolic acids, 340 nm for flavones, and 360 nm for flavonol glycosides.

Quantification was performed using external calibration curves constructed with authentic reference compounds. When authentic standards corresponding directly to the identified compounds were unavailable, structurally related reference compounds were used for quantification based on similarities in chemical structure or functional groups. Results were expressed as mg/L of beverage. All analyses were performed in triplicate.

### 3.6. Analysis of Volatile Organic Compounds

Volatile organic compounds (VOCs) were analyzed by headspace solid-phase microextraction Arrow coupled with gas chromatography–mass spectrometry (HS-SPME Arrow-GC/MS), based on the method described by Łyczko et al. [44]. Ethanol was determined separately using the analytical method described above and was not included in the volatile alcohol concentrations reported in Table 4. Analyses were performed using a Shimadzu GCMS-QP2020 Plus system (Shimadzu, Kyoto, Japan) equipped with an SH-Rxi-5Sil MS capillary column (30 m × 0.25 mm × 0.25 µm; Shimadzu, Kyoto, Japan). Volatile compounds were quantified based on peak areas relative to an internal standard. The results were expressed as ng/mL of beverage. All measurements were performed in triplicate.

### 3.7. Antioxidant and Biological Activities

The antioxidant capacity and enzyme-inhibitory activities of the fermented gooseberry juices were determined using a Synergy H1 microplate reader (BioTek Instruments, Inc., Winooski, VT, USA), according to the procedures described by Chandran et al. (2025) [41]. Antioxidant capacity was assessed using the ABTS, DPPH, and ferric reducing antioxidant power (FRAP) assays, with the results expressed as µmol Trolox equivalents (TE)/100 mL.

Cyclooxygenase-inhibitory activity was evaluated by measuring the inhibition of COX-1 and COX-2. The inhibitory effects against carbohydrate-hydrolyzing enzymes were assessed using α-amylase and α-glucosidase assays, whereas cholinesterase-inhibitory activity was determined by measuring the inhibition of acetylcholinesterase (AChE) and butyrylcholinesterase (BuChE) using Ellman’s colorimetric assay. Enzyme-inhibitory activities were expressed as percentage inhibition relative to the corresponding enzyme activity. All analyses were performed in triplicate.

### 3.8. Cell Culture and In Vitro Assays

#### 3.8.1. Cell Culturing of HepG2

In this study, HepG2 hepatocellular carcinoma cells were used to model disrupted liver homeostasis. Cells were maintained in MEM medium containing 2 mM glutamine, 10% fetal bovine serum, penicillin (1 U/mL), and streptomycin (1 µg/mL). Cells were maintained at 37 °C with 5% CO_2_ and seeded at 4–5 × 10^5^ cells per 10-cm dish. Subculturing was performed at 80% confluency using PBS washes and 5 min of trypsinization, after which cells were reseeded into new plates at approximately 30% confluency.

#### 3.8.2. Preparation of Polyphenolic Extracts and Treatment of HepG2 Cells

Polyphenolic extracts were prepared from red and white gooseberry-based beverages supplemented with sucrose or buckwheat honey and collected after 10 days of SCOBY fermentation. For each beverage formulation, 500 mL of the fermented sample was subjected to column chromatography using Amberlite XAD-16 resin. The column was washed with distilled water to reduce the content of water-soluble matrix constituents, particularly sugars and organic acids. The column effluent was monitored refractometrically (°Brix), and washing was continued until a reading of 0 °Brix was obtained. The compounds retained on the resin were subsequently eluted with 96% (*v*/*v*) ethanol. The collected ethanolic eluate was concentrated under reduced pressure at 40 °C, frozen, and lyophilized to obtain dried preparations enriched in phenolic compounds. These preparations were subsequently used in the cell-based assays.

The extracts were dissolved in dimethyl sulfoxide (DMSO) to prepare 50 mg/mL stocks. Cells were seeded a day prior to treatment and then exposed for 4 days to vehicle (DMSO-Control) or test extracts at final concentrations of 25–400 µg/mL. Polyphenolic extracts used for the cell culture experiments were prepared from beverages collected after 10 days of fermentation.

#### 3.8.3. Harvesting and Counting of Cells

After treatment, the cells were harvested and counted using trypan blue and a hemocytometer to assess the number of viable and dead cells. Cell growth was calculated as a percentage of viable cells after treatment with an extract relative to control. Cell viability was determined based on a formula [viable/(viable + dead) cell number)] × 100% for each condition with no normalization. The IC25 and IC50 values (i.e., extract concentrations leading to a 25% and 50% cell growth reduction, respectively, relative to control while maintaining at least 90% viability) were calculated to compare potency. Cell pellets were stored at −80 °C for RNA isolation.

#### 3.8.4. RNA Isolation and Quantitative Reverse Transcriptase Polymerase Chain Reaction (qRT-PCR)

Total RNA from cell pellets was isolated using TRIzol (Invitrogen, Thermo Fisher Scientific, Waltham, MA, USA), according to the manufacturer’s protocol, followed by treatment with amplification-grade DNase I (Invitrogen, Thermo Fisher Scientific, Waltham, MA, USA) to remove residual genomic DNA prior to cDNA synthesis. To convert the isolated RNA into cDNA for qRT-PCR, a 10× dilution of random primers was added to 1 µg of purified RNA and the mixture was incubated at 70 °C for 5 min. AMV buffer, RNase inhibitors, dNTPs (10 μM), and AMV reverse transcriptase were added to the tube and incubated according to manufacturer’s directions. The product was then diluted with double distilled H_2_O for quantitative PCR.

Genes of interest for qRT-PCR were selected based on RNA sequencing data from the Stefanska Nutriepigenomics Laboratory on livers from a rat model of liver cancer where the diet was supplemented with polyphenols, specifically pterostilbene [24,45]. The genes of interest selected were: *G6PC*, *FABP4*, *ALDH1L1*, *WNT10B*, *EPCAM* and *SERPINE1*. CFX96 Touch Real-Time PCR Detection System (Bio-Rad Laboratories, Hercules, CA, USA) was used to amplify target sequences using 2 µL of cDNA as a template, 400 nM forward and reverse primers, and 10 µL of SsoFast EvaGreen Supermix (Bio-Rad Laboratories, Hercules, CA, USA) in a final volume of 20 µL. The amplification reaction included denaturation at 95 °C for 10 min, amplification for 60 cycles at 95 °C for 10 s, annealing temperature for 10 s, 72 °C for 10 s, and final extension at 72 °C for 10 min. To quantify mRNA levels of the tested genes, the ∆CT method using a reference gene (*GAPDH*) was used, which is a variation of the Livak method. The results are presented as gene of interest/*GAPDH*, based on the formula 2^(-[C_T (target)-C_T (reference)]) [45].

### 3.9. Sensory Evaluation

Sensory evaluation was conducted as a consumer acceptance test using a 9-point hedonic scale (1 = dislike extremely, 9 = like extremely). Six samples were evaluated: unfermented red and white gooseberry juices without added sweetener or kombucha starter, and four beverages obtained after 10 days of fermentation (D_10), prepared from red or white gooseberry juice supplemented with either sucrose or buckwheat honey. The evaluated attributes included taste, aroma, sweetness, acidity, and overall acceptability. The evaluation was conducted in the Sensory Analysis Laboratory at the Faculty of Biotechnology and Food Science, Wrocław University of Environmental and Life Sciences, Poland. The panel consisted of 20 untrained adult consumers (university students) recruited through announcements published on the university website and campus notice boards. Each participant was provided with a 30 mL serving of each beverage. The samples were coded with random three-digit numbers, served at 23 °C under controlled conditions, and presented in randomized order. All participants were adults and were informed about the presence of alcohol in the fermented samples before the evaluation. Assessments were recorded using a structured evaluation sheet.

The sensory study involving human participants was reviewed and approved by the Ethics Committee for Scientific Research at Wrocław University of Environmental and Life Sciences (approval No. N0N00000.0020.1.10.2.2025, issued on 14 July 2025). Participation was voluntary, and all participants provided informed consent prior to participation and were informed of their right to withdraw from the study at any time. Before sensory evaluation, all participants were informed about the nature of the study, the presence of alcohol in the fermented beverages, and potential allergens.

### 3.10. Statistical Analysis

Statistical analyses were performed using Statistica 13.1 software (StatSoft Inc., Tulsa, OK, USA). Unless otherwise stated, results are expressed as mean ± standard deviation (SD) of three independent fermentations. Technical replicates were averaged within each independent fermentation before statistical analysis.

For physicochemical, phytochemical, and in vitro functional parameters measured during fermentation, data were analyzed using factorial repeated-measures analysis of variance (ANOVA), with juice type (red or white) and carbon source (sucrose or honey) as between-subject factors and fermentation time (D_0, D_5, and D_10) as the within-subject repeated factor. Main effects and interactions between juice type, carbon source, and fermentation time were evaluated. When significant effects were detected, differences between individual means were assessed using Duncan’s multiple range test or Tukey HSD test. Statistical significance was set at *p* < 0.05.

## 4. Conclusions

SCOBY fermentation substantially modified the chemical composition and functional properties of gooseberry-based beverages, resulting in sugar depletion, ethanol and organic acid accumulation, and extensive remodeling of the polyphenolic and volatile profiles. These compositional changes were accompanied by increased antioxidant capacity and selected enzyme inhibitory activities, although the magnitude of the response depended on the gooseberry matrix and carbon source. In particular, inhibition of α-amylase and α-glucosidase remained low and does not support conclusions regarding antidiabetic efficacy.

Red gooseberry-based beverages exhibited higher total phenolic content and generally stronger antioxidant and enzyme inhibitory activities, with particularly high values observed for the honey-supplemented variant. However, despite these compositional and functional characteristics, polyphenolic extracts derived from white gooseberry fermentation products showed a stronger inhibitory effect on HepG2 cell growth than those derived from red gooseberry. This divergence demonstrates that the cellular effects of the extracts cannot be predicted from total phenolic content or antioxidant capacity alone and suggests that the qualitative composition of the phenolic fraction and fermentation-induced transformations may be important determinants of biological activity. Gene expression analysis of WGJ-derived extracts further revealed compound-dependent modulation of genes involved in metabolic homeostasis and oncogenic signaling, indicating a complex cellular response rather than a uniformly beneficial molecular effect.

Sensory evaluation provided an additional perspective on the formulation-dependent effects of fermentation. The most favorable overall sensory profile was observed for sucrose-supplemented fermented red gooseberry juice, whereas the honey-supplemented RGJ variant showed particularly high antioxidant capacity and enzyme inhibitory activities. The use of a common black tea-derived starter without a separate starter-specific control limits the attribution of individual phenolic compounds and associated biological activities exclusively to the gooseberry matrix or fermentation-induced transformations. This divergence indicates that maximizing functional characteristics does not necessarily result in the highest consumer acceptance and should be considered when designing fermented fruit beverages.

In summary, the present study demonstrates that SCOBY fermentation is an effective approach for extensively modifying the chemical and functional characteristics of gooseberry-based beverages. Importantly, the effects were strongly dependent on both the fruit matrix and carbon source, and chemical composition, in vitro biological activity, cellular response, and sensory acceptance did not necessarily follow the same pattern. These findings highlight the importance of integrating compositional, biological, and sensory analyses when evaluating and optimizing fruit-based fermented beverages.

## Figures and Tables

**Table 1:** Total viable count (TVC), lactic acid bacteria (LAB), acetic acid bacteria (AAB), and yeast/mold counts in kombucha beverages after inoculation (start) and on day 10 of fermentation (expressed as CFU/mL).

Samples	Total Viable Account (TVC)		Lactic Acid Bacteria (LAB)		Acetic Acid Bacteria (AAB)		Yeast and Filamentous Fungi	
	D_0	D_10	D_0	D_10	D_0	D_10	D_0	D_10
RGJ-S	6.0 × 10^3^	4.1 × 10^4^	<10^2^ *	4.4 × 10^5^	<10^2^ *	1.0 × 10^2^	6.0 × 10^3^	1.6 × 10^5^
RGJ-H	3.2 × 10^3^	4.0 × 10^4^	<10^2^ *	3.5 × 10^5^	<10^2^ *	<10^2^ *	5.0 × 10^3^	1.5 × 10^5^
WGJ-S	2.1 × 10^2^	1.0 × 10^3^	<10^2^ *	1.9 × 10^4^	<10^2^ *	3.9 × 10^2^	2.5 × 10^3^	3.5 × 10^3^
WGJ-H	4.8 × 10^3^	7.3 × 10^3^	<10^2^ *	4.2 × 10^4^	<10^2^ *	<10^2^ *	3.3 × 10^3^	1.6 × 10^4^

* <10^2^ indicates counts below the lowest quantifiable level under the applied enumeration conditions; Abbreviations: WGJ_S—white gooseberry juice with sucrose; WGJ_H—white gooseberry juice with honey; RGJ_S—red gooseberry juice with sucrose; RGJ_H—red gooseberry juice with honey.

**Table 2:** Changes in the basic chemical composition and physicochemical properties of red and white gooseberry-based beverages during 10 days of SCOBY fermentation with sucrose or buckwheat honey as the added carbon source.

		TSS [°Brix]	pH	Dry Matter [%]	Ethanol [%]	Viscosity [mPas]	Organic Acids [g/100 mL]			Sugars [g/L]		
							Malic Acid	Lactic Acid	Acetic Acid	Fructose	Glucose	Sucrose
WGJ_S	D_0	14.40 ± 0.02 ^a^	3.03 ± 0.01 ^a^	15.31 ± 0.12 ^a^	0.00 ± 0.00 ^c^	7.41 ± 0.11 ^b^	1.34 ± 0.02 ^c^	0.01 ± 0.00 ^c^	0.00 ± 0.00 ^b^	22.63 ± 0.33 ^b^	28.29 ± 0.51 ^c^	89.05 ± 0.51 ^a^
	D_5	9.00 ± 0.01 ^e^	2.77 ± 0.02 ^c^	8.87 ± 0.09 ^f^	0.06 ± 0.00 ^c^	4.47 ± 0.09 ^e^	1.42 ± 0.01 ^b^	0.16 ± 0.01 ^b^	0.00 ± 0.00 ^b^	1.35 ± 0.00 ^f^	6.53 ± 0.32 ^f^	52.69 ± 0.40 ^c^
	D_10	5.80 ± 0.02 ^g^	2.73 ± 0.01 ^c^	4.09 ± 0.08 ^i^	6.28 ± 0.05 ^a^	3.98 ± 0.05 ^f^	1.68 ± 0.05 ^a^	0.68 ± 0.01 ^a^	3.03 ± 0.20 ^a^	0.00 ± 0.00 ^g^	0.42 ± 0.00 ^h^	9.88 ± 0.10 ^e^
WGJ_H	D_0	13.80 ± 0.3 ^bc^	2.86 ± 0.03 ^b^	14.34 ± 0.10 ^b^	0.00 ± 0.00 ^c^	5.17 ± 0.08 ^d^	1.32 ± 0.02 ^c^	0.01 ± 0.00 ^c^	0.00 ± 0.00 ^b^	62.63 ± 0.19 ^a^	54.02 ± 1.02 ^a^	6.64 ± 0.13 ^f^
	D_5	11.30 ± 0.02 ^cd^	2.84 ± 0.02 ^b^	12.28 ± 0.11 ^c^	0.04 ± 0.00 ^c^	5.04 ± 0.11 ^d^	1.45 ± 0.04 ^b^	0.21 ± 0.00 ^b^	0.00 ± 0.00 ^b^	17.31 ± 0.21 ^d^	33.62 ± 0.27 ^b^	0.24 ± 0.01 ^g^
	D_10	9.10 ± 0.01 ^e^	2.81 ± 0.01 ^bc^	8.14 ± 0.05 ^g^	4.76 ± 0.02 ^b^	0.90 ± 0.06 ^g^	1.66 ± 0.05 ^a^	0.66 ± 0.02 ^a^	2.51 ± 0.08 ^a^	0.00 ± 0.00 ^g^	12.88 ± 0.16 ^de^	0.00 ± 0.00 ^h^
RGJ_S	D_0	12.80 ± 0.03 ^c^	2.96 ± 0.02 ^a^	15.01 ± 0.09 ^e^	0.00 ± 0.00 ^c^	7.40 ± 0.10 ^b^	1.25 ± 0.03 ^c^	0.01 ± 0.00 ^c^	0.00 ± 0.00 ^b^	22.49 ± 0.12 ^b^	30.62 ± 0.25 ^b^	89.10 ± 0.42 ^a^
	D_5	10.40 ± 0.02 ^d^	2.83 ± 0.01 ^bc^	9.89 ± 0.10 ^e^	0.07± 0.00 ^c^	7.41 ± 0.04 ^b^	1.39 ± 0.01 ^bc^	0.18 ± 0.01 ^b^	0.00 ± 0.00 ^b^	14.98 ± 0.04 ^e^	14.44 ± 0.13 ^d^	61.25 ± 0.265 ^b^
	D_10	8.00 ± 0.01 ^f^	2.81 ± 0.02 ^bc^	5.31 ± 0.04 ^h^	4.27 ± 0.01 ^b^	4.48 ± 0.06 ^e^	1.78 ± 0.03 ^a^	0.72 ± 0.01 ^a^	2.20 ± 0.02 ^a^	0.02 ± 0.00 ^g^	5.89 ± 0.10 ^f^	12.05 ± 0.59 ^d^
RGJ_H	D_0	10.50 ± 0.03 ^d^	3.02 ± 0.03 ^a^	14.21 ± 0.11 ^d^	0.00 ± 0.00 ^c^	7.83 ± 0.11 ^a^	1.25 ± 0.02 ^c^	0.01 ± 0.00 ^c^	0.00 ± 0.00 ^b^	62.86 ± 0.11 ^a^	55.87 ± 0.11 ^a^	6.77 ± 0.02 ^f^
	D_5	9.40 ± 0.02 ^e^	2.85 ± 0.01 ^b^	8.46 ± 0.10 ^fg^	0.03 ± 0.00 ^c^	6.45 ± 0.09 ^c^	1.39 ± 0.04 ^bc^	0.21 ± 0.00 ^b^	0.00 ± 0.00 ^b^	20.16 ± 0.02 ^c^	11.87 ± 0.13 ^de^	0.30 ± 0.00 ^g^
	D_10	8.40 ± 0.01 ^f^	2.82 ± 0.02 ^bc^	5.17 ± 0.03 ^h^	4.27 ± 0.02 ^b^	5.44 ± 0.07 ^d^	1.52 ± 0.03 ^ab^	0.69 ± 0.02 ^a^	2.61 ± 0.02 ^a^	0.02 ± 0.00 ^g^	1.36 ± 0.02 ^g^	0.00 ± 0.00 ^h^

Values are presented as mean ± standard deviation (SD) of three independent fermentations (*n* = 3). Different superscript letters within the same parameter indicate statistically significant differences between means (*p* < 0.05). Abbreviations: TSS—total soluble solids; WGJ_S—white gooseberry juice with sugar; WGJ_H—white gooseberry juice with honey; RGJ_S—red gooseberry juice with sugar; RGJ_H—red gooseberry juice with honey; D_0—day 0 of fermentation; D_5—day 5 of fermentation; D_10—day 10 of fermentation.

**Table 3:** Changes in major phenolic groups in red and white gooseberry-based beverages during SCOBY fermentation (mg/L).

		Phenolic Acids	Theaflavins	Flavan-3-ols and Proanthocyanidins	Anthocyanins	Flavonols	Total
WGJ_S	D_0	459.82 ± 15.75 ^de^	0.39 ± 0.02 ^e^	256.15 ± 9.22 ^f^	nd	60.88 ± 2.57 ^d^	777.24 ± 27.52 ^f^
	D_5	460.00 ± 20.60 ^de^	0.53 ± 0.07 ^d^	424.65 ± 13.08 ^e^	nd	54.84 ± 2.69 ^e^	940.02 ± 36.26 ^e^
	D_10	440.39 ± 14.77 ^d^	0.67 ± 0.13 ^bc^	499.67 ± 16.60 ^d^	nd	40.19 ± 1.38 ^f^	980.92 ± 32.88 ^e^
WGJ_H	D_0	461.45 ± 16.90 ^de^	0.39 ± 0.01 ^e^	257.8 ± 11.21 ^f^	nd	64.25 ± 2.32 ^d^	783.89 ± 30.51 ^f^
	D_5	472.01 ± 18.35 ^d^	0.56 ± 0.02 ^d^	430.69 ± 17.76 ^e^	nd	55.31 ± 1.95 ^e^	958.57 ± 38.24 ^e^
	D_10	483.47 ± 16.44 ^d^	0.73 ± 0.01 ^b^	549.61 ± 29.57 ^c^	nd	42.15 ± 1.53 ^f^	1075.96 ± 47.56 ^d^
RGJ_S	D_0	647.29 ± 19.99 ^b^	0.66 ± 0.09 ^bc^	446.03 ± 21.60 ^e^	22.68 ± 0.93 ^a^	97.67 ± 5.19 ^a^	1214.33 ± 47.75 ^c^
	D_5	579.97 ± 15.59 ^c^	0.70 ± 0.01 ^b^	538.43 ± 20.05 ^c^	23.66 ± 0.87 ^a^	80.88 ± 3.32 ^b^	1223.64 ± 39.79 ^c^
	D_10	656.30 ± 27.56 ^b^	0.63 ± 0.02 ^c^	935.06 ± 35.51 ^b^	24.34 ± 0.73 ^a^	61.18 ± 2.38 ^d^	1677.51 ± 66.10 ^b^
RGJ_H	D_0	650.11 ± 34.56 ^b^	0.67 ± 0.02 ^bc^	445.61 ± 12.57 ^e^	22.71 ± 0.84 ^a^	101.79 ± 4.73 ^a^	1220.89 ± 52.65 ^c^
	D_5	598.93 ± 25.41 ^c^	0.63 ± 0.03 ^c^	535.15 ± 22.78 ^c^	24.89 ± 1.01 ^a^	81.38 ± 3.81 ^b^	1240.98 ± 52.81 ^c^
	D_10	707.03 ± 28.98 ^a^	0.80 ± 0.02 ^a^	1046.48 ± 53.53 ^a^	26.56 ± 1.70 ^a^	78.82 ± 3.00 ^c^	1859.69 ± 87.21 ^a^

Values are presented as mean ± standard deviation (SD) of three independent fermentations (*n* = 3). Different superscript letters within the same parameter indicate statistically significant differences between means (*p* < 0.05). Abbreviations: WGJ_S—white gooseberry juice with sucrose; WGJ_H—white gooseberry juice with honey; RGJ_S—red gooseberry juice with sucrose; RGJ_H—red gooseberry juice with honey; D_0—day 0 of fermentation; D_5—day 5 of fermentation; D_10—day 10 of fermentation; nd—not detected.

**Table 4:** Changes in major volatile compound groups, excluding ethanol, in red and white gooseberry-based beverages during SCOBY fermentation (ng/mL).

		Esters	Alcohols	Acids	Aldehydes	Ketones	Terpenes & Terpenoids	Furans & Furan Derivatives	Total
WGJ_S	D_0	895.01 ± 23.58 ^e^	2667.77 ± 45.18 ^d^	597.05 ± 20.36 ^a^	121.19 ± 5.87 ^d^	3.87 ± 0.13 ^c^	995.31 ± 18.26 ^a^	nd	5280.20 ± 58.87 ^c^
	D_5	598.46 ± 11.89 ^f^	1787.63 ± 39.85 ^e^	399.68 ± 21.15 ^b^	80.57 ± 7.12 ^e^	2.59 ± 0.09 ^c^	660.98 ± 20.15 ^c^	nd	3529.91 ± 47.63 ^d^
	D_10	597.07 ± 14.46 ^f^	1783.78 ± 29.29 ^e^	398.82 ± 12.54 ^b^	80.59 ± 6.84 ^e^	2.59 ± 0.11 ^c^	660.3 ± 11.45 ^c^	nd	3523.15± 33.30 ^d^
WGJ_H	D_0	277.64 ± 11.50 ^g^	4293.84 ± 58.79 ^b^	12.81 ± 0.23 ^e^	274.75 ± 11.21 ^b^	66.09 ± 2.32 ^b^	805.20 ± 25.87 ^b^	489.32 ± 14.78 ^a^	6219.65 ± 87.46 ^b^
	D_5	273.78 ± 14.89 ^g^	4290.81 ± 31.42 ^b^	13.28 ± 0.28 ^e^	272.3 ± 10.08 ^b^	65.95 ± 3.41 ^b^	798.96 ± 32.56 ^b^	485.73 ± 17.99 ^a^	6200.81 ± 90.01 ^b^
	D_10	271.96 ± 10.12 ^g^	4267.65 ± 66.01 ^c^	12.73 ± 0.31 ^e^	268.44 ± 15.32 ^b^	65.72 ± 2.65 ^b^	796.77 ± 40.41 ^b^	484.42± 13.67 ^a^	6167.69 ± 49.30 ^b^
RGJ_S	D_0	896.76 ± 23.23 ^e^	364.87 ± 14.29 ^g^	75.08 ± 1.25 ^d^	115.57 ± 10.52 ^d^	nd	291.05 ± 15.33 ^e^	nd	1743.33 ± 12.88 ^f^
	D_5	1370.56 ± 35.60 ^a^	550.76 ± 11.83 ^f^	115.48 ± 5.28 ^c^	181.47 ± 11.33 ^c^	nd	449.24 ± 11.28 ^d^	nd	2667.51 ± 25.89 ^e^
	D_10	1349.78 ± 21.08 ^b^	549.02 ± 19.32 ^f^	112.59 ± 4.63 ^c^	173.31 ± 14.85 ^c^	nd	438.96 ± 19.56 ^d^	nd	2623.66 ± 31.03 ^e^
RGJ_H	D_0	1007.23 ± 15.89 ^c^	6392.59 ± 85.23 ^a^	nd	323.97 ± 13.58 ^a^	681.71 ± 6.88 ^a^	137.42 ± 10.23 ^f^	nd	8542.93 ± 52.87 ^a^
	D_5	997.66 ± 11.33 ^d^	6349.73 ± 56.20 ^b^	nd	323.92 ± 20.07 ^a^	675.95 ± 11.52 ^a^	139.07 ± 8.75 ^f^	nd	8486.33 ± 63.87 ^a^
	D_10	992.62 ± 17.18 ^d^	6390.80 ± 67.39 ^a^	nd	315.63 ± 15.36 ^a^	686.57 ± 14.56 ^a^	133.84 ± 12.69 ^f^	nd	8519.45 ± 78.03 ^a^

Values are presented as mean ± standard deviation (SD) of three independent fermentations (*n* = 3). Different superscript letters within the same parameter indicate statistically significant differences between means (*p* < 0.05). Abbreviations: WGJ_S—white gooseberry juice with sucrose; WGJ_H—white gooseberry juice with honey; RGJ_S—red gooseberry juice with sucrose; RGJ_H—red gooseberry juice with honey; D_0—day 0 of fermentation; D_5—day 5 of fermentation; D_10—day 10 of fermentation; nd—not detected.

**Table 5:** Changes in antioxidant capacity and enzyme inhibitory activities of red and white gooseberry-based beverages during SCOBY fermentation.

		DPPH	ABTS	FRAP	COX1	COX2	AChE	BuChE	a-Amylase	a-Glucosidase
		[µMol Tx/100 mL]			Inhibition [%]					
WGJ_S	D_0	100.27 ± 1.21 ^g^	144.38 ± 1.32 ^f^	190.44 ± 2.01 ^f^	65.25 ± 0.98 ^de^	33.15 ± 0.52 ^e^	9.28 ± 0.09 ^h^	11.89 ± 0.08 ^e^	2.05 ± 0.02 ^f^	7.18 ± 0.02 ^f^
	D_5	105.33 ± 1.42 ^fg^	154.41 ± 1.52 ^e^	240.26 ± 2.15 ^e^	67.59 ± 1.02 ^d^	34.41 ± 0.47 ^e^	10.06 ± 0.11 ^g^	12.56 ± 0.03 ^e^	2.13 ± 0.01 ^f^	7.52 ± 0.05 ^f^
	D_10	111.03 ± 1.26 ^f^	160.10 ± 1.18 ^de^	248.80 ± 2.36 ^e^	69.96 ± 1.11 ^d^	36.09 ± 0.32 ^de^	11.90 ± 0.08 ^f^	18.00 ± 0.10 ^d^	3.04 ± 0.02 ^e^	7.88 ± 0.03 ^ef^
WGJ_H	D_0	107.27 ± 1.13 ^f^	154.36 ± 1.21 ^e^	241.26 ± 2.14 ^e^	73.58 ± 0.88 ^c^	39.84 ± 0.58 ^d^	14.82 ± 0.12 ^e^	15.12 ± 0.05 ^de^	5.12 ± 0.02 ^b^	9.12 ± 0.03 ^d^
	D_5	111.27 ± 1.11 ^f^	167.79 ± 1.31 ^d^	254.03 ± 2.09 ^e^	77.84 ± 0.89 ^c^	40.99 ± 0.69 ^cd^	16.16 ± 0.10 ^cd^	19.22 ± 0.08 ^cd^	5.36 ± 0.01 ^b^	9.68 ± 0.02 ^cd^
	D_10	121.04 ± 1.45 ^e^	182.04 ± 1.08 ^c^	329.11 ± 2.25 ^c^	81.12 ± 1.03 ^b^	42.15 ± 0.41 ^c^	21.50 ± 0.09 ^b^	20.25 ± 0.10 ^c^	6.01 ± 0.03 ^a^	10.03 ± 0.04 ^c^
RGJ_S	D_0	135.70 ± 1.29 ^d^	189.43 ± 1.54 ^c^	304.23 ± 2.56 ^d^	80.24 ± 1.13 ^bc^	40.58 ± 0.35 ^cd^	15.85 ± 0.12 ^d^	17.69 ± 0.11 ^d^	3.63 ± 0.02 ^d^	7.99 ± 0.02 ^e^
	D_5	140.37 ± 1.58 ^cd^	195.39 ± 1.11 ^bc^	306.01 ± 2.58 ^d^	82.66 ± 1.20 ^b^	41.08 ± 0.48 ^c^	17.00 ± 0.13 ^c^	19.03 ± 0.06 ^cd^	3.99 ± 0.03 ^d^	8.24 ± 0.04 ^e^
	D_10	147.27 ± 1.01 ^c^	218.84 ± 1.39 ^b^	313.38 ± 2.90 ^cd^	83.13 ± 0.78 ^b^	45.25 ± 0.29 ^bc^	21.36 ± 0.12 ^b^	22.14 ± 0.08 ^c^	4.24 ± 0.01 ^c^	8.99 ± 0.05 ^d^
RGJ_H	D_0	145.26 ± 1.26 ^c^	202.35 ± 1.52 ^bc^	320.51 ± 2.13 ^c^	87.72 ± 1.12 ^ab^	46.98 ± 0.31 ^ab^	22.89 ± 0.09 ^b^	29.56 ± 0.12 ^b^	5.54 ± 0.01 ^b^	11.06 ± 0.03 ^b^
	D_5	151.23 ± 1.42 ^b^	212.02 ± 1.32 ^b^	361.80 ± 3.08 ^b^	89.09 ± 2.07 ^a^	48.08 ± 0.12 ^ab^	29.65 ± 0.14 ^ab^	32.65 ± 0.09 ^ab^	5.88 ± 0.02 ^ab^	11.87 ± 0.08 ^a^
	D_10	165.37 ± 1.17 ^a^	251.36 ± 1.02 ^a^	451.22 ± 2.54 ^a^	92.99 ± 0.69 ^a^	52.12 ± 0.28 ^a^	35.18 ± 0.11 ^a^	36.19 ± 0.10 ^a^	6.25 ± 0.02 ^a^	12.08 ± 0.01 ^a^

Values are presented as mean ± standard deviation (SD) of three independent fermentations (*n* = 3). Enzyme inhibition values correspond to assays performed using undiluted juice samples. Different superscript letters within the same parameter indicate statistically significant differences between means (*p* < 0.05). Abbreviations: WGJ_S—white gooseberry juice with sucrose; WGJ_H—white gooseberry juice with honey; RGJ_S—red gooseberry juice with sucrose; RGJ_H—red gooseberry juice with honey; D_0—day 0 of fermentation; D_5—day 5 of fermentation; D_10—day 10 of fermentation.

## Data Availability

The original contributions presented in this study are included in the article. Further inquiries can be directed to the corresponding author.

## Supplementary Materials

The following supporting information can be downloaded at https://www.mdpi.com/article/10.3390/ijms27219794/s1.

## Abbreviations

The following abbreviations are used in this manuscript:

WGJ_S	White Gooseberry Juice with Sugar.
RGJ_S	Red: Gooseberry Juice with Sugar.
WGJ_H	White: Gooseberry Juice with Honey.
RGJ_H	Red: Gooseberry Juice with Honey.
TVC	Total viable count
LAB	Lactic acid bacteria
AAB	Acetic acid bacteria
UPLC	Ultra-Performance Liquid Chromatography
PDA	Photodiode Array
Q-ToF-MS	Quadrupole Time-of-Flight Mass Spectrometry
DPPH	2,2-Diphenyl-1-picrylhydrazyl
ABTS	2,2′-Azino-bis(3-ethylbenzothiazoline-6-sulfonic acid)
FRAP	Ferric Reducing Antioxidant Power
COX-1	Cyclooxygenase-1
COX-2	Cyclooxygenase-2
AChE	Acetylcholinesterase
BuChE	Butyrylcholinesterase
BEH	Ethylene Bridged Hybrid
C18	Octadecylsilane (C18) stationary phase
ESI	Electrospray Ionization
VOC	Volatile organic compounds
SCOBY	Symbiotic Culture of Bacteria and Yeast
HepG2	Human hepatocellular carcinoma cell line HepG2
MEM	Minimum Essential Medium
FBS	Fetal Bovine Serum
PBS	Phosphate-Buffered Saline
DMSO	Dimethyl Sulfoxide
*G6PC*	Glucose-6-Phosphatase Catalytic Subunit 1
*FABP4*	Fatty Acid Binding Protein 4
*ALDH1L1*	Aldehyde Dehydrogenase 1 Family Member L1
*WNT10B*	Wnt Family Member 10B
*EPCAM*	Epithelial Cell Adhesion Molecule
*SERPINE1*	Serpin Family E Member 1
*GAPDH*	Glyceraldehyde-3-Phosphate Dehydrogenase
PCR	Polymerase Chain Reaction
cDNA	Complementary DNA
mRNA	Messenger RNA
ΔCT	Delta Cycle Threshold
