# Supplementary material for: Kombucha Fermentation Remodels the Phytochemical Composition of Gooseberry Juices and Reveals a Divergence Between Phenolic Abundance and HepG2 Cell Growth Inhibition
Source: Int J Mol Sci. 2026 Oct 31;27(21):9794. doi: 10.3390/ijms27219794 (PMC13762948; doi:10.3390/ijms27219794)
Supplement: Supplementary file 1 [file ijms-27-09794-s001.zip › ijms-4625360-supplementary.pdf]

Table S1. Changes in individual phenolic compounds in red and white gooseberry-based beverages during SCOBY fermentation (mg/L).

			WGJ_S			WGJ_H			RGJ_S			RGJ_H		
MS	MS/MS	Compound	D0	D5	D10	D0	D5	D10	D0	D5	D10	D0	D5	D10
193.0186		Ferulic acid	3.47 ± 0.07	3.07 ± 0.14	2.51 ± 0.13	3.42 ± 0.1	2.98 ± 0.09	2.66 ± 0.14	4.06 ± 0.15	3.45 ± 0.16	3.4 ± 0.15	4.07 ± 0.2	3.78 ± 0.22	3.6 ± 0.25
373.0242	179	Syringic acid derivative	1.05 ± 0.03	0.31 ± 0.02	nd	1 ± 0.02	0.56 ± 0.03	0.22 ± 0.01	0.92 ± 0.05	0.67 ± 0.03	0.59 ± 0.02	0.98 ± 0.05	0.86 ± 0.02	0.77 ± 0.03
295.0038	191.0038	Caffeoyl malate	28.79 ± 1.24	25.51 ± 1.55	24.94 ± 1.1	29.01 ± 1.11	26.16 ± 1.1	25.22 ± 0.58	69.81 ± 3.79	61.29 ± 3.31	58.9 ± 4.11	69.37 ± 1.84	61.85 ± 1.92	59.03 ± 3.82
191.0017	175/132/114	Quinic acid derivative	3.79 ± 0.26	4.17 ± 0.28	4.77 ± 0.16	3.7 ± 0.16	3.98 ± 0.14	4.2 ± 0.17	5.15 ± 0.05	6.34 ± 0.34	7.28 ± 0.11	5.19 ± 0.08	6.23 ± 0.18	7.31 ± 0.49
295.0038	191.0038	Caffeoyl malate	179.57 ± 6.54	158.83 ± 8.61	135.33 ± 5.81	180.25 ± 7.93	165.12 ± 7.35	148.55 ± 6.4	309.01 ± 5.1	279.6 ± 2.94	244.15 ± 11.87	305.42 ± 20.52	283.22 ± 12.52	247.23 ± 4.75
341.0788	190.9996	Caffeoyhexose	nd	nd	nd	nd	nd	nd	6.26 ± 0.17	1.97 ± 0.03	1.2 ± 0.01	6.48 ± 0.44	2.94 ± 0.15	nd
353.0513	172	Caffeoylquinic acid	8.24 ± 0.14	6.71 ± 0.41	6.22 ± 0.33	8.35 ± 0.1	7.17 ± 0.19	6.98 ± 0.26	9.89 ± 0.24	5.09 ± 0.3	4.33 ± 0.25	9.29 ± 0.4	5.06 ± 0.11	3.4 ± 0.1
191.0032		Quinic acid	nd	9.11 ± 0.35	11.52 ± 0.31	nd	8.54 ± 0.31	16.24 ± 0.17	nd	0.76 ± 0.03	1.12 ± 0.04	nd	1.53 ± 0.1	1.73 ± 0.08
353.051	191/179	Caffeoylquinic acid	2.84 ± 0.12	1.71 ± 0.03	1.51 ± 0.09	2.9 ± 0.2	1.85 ± 0.04	1.64 ± 0.08	2.95 ± 0.2	1.68 ± 0.11	0.88 ± 0.05	3.16 ± 0.15	1.14 ± 0.02	0.24 ± 0.01
191.0028	173.0096/127.9557	Quinic acid derivative	22.54 ± 0.29	6.41 ± 0.41	0.52 ± 0.02	22.57 ± 0.46	15.21 ± 0.16	2.36 ± 0.14	13.01 ± 0.76	10.15 ± 0.21	2.77 ± 0.07	13.07 ± 0.42	4.5 ± 0.2	2.44 ± 0.04
385.0256	223.0031/164.5872	Sinapic acid hexoside	1.77 ± 0.07	1.59 ± 0.08	0.56 ± 0.03	1.82 ± 0.04	1.48 ± 0.05	1.01 ± 0.01	1.4 ± 0.06	0.73 ± 0.02	0.16 ± 0.01	1.36 ± 0.02	1.03 ± 0.02	0.93 ± 0.03
353.0473	191	Caffeoylquinic acid	17.18 ± 0.4	7.49 ± 0.45	4.72 ± 0.26	16.98 ± 0.62	9.25 ± 0.33	5.29 ± 0.21	23.08 ± 1.35	14.56 ± 0.53	7 ± 0.43	23.69 ± 1.41	16.88 ± 1.16	5.6 ± 0.36
457.0714	305.0655/169.0118	Gallocatechin gallate (GCG) unknown isomer	54.52 ± 1.3	100.06 ± 3.68	114.14 ± 1.24	52.87 ± 2.74	86.79 ± 3.38	120.47 ± 2.69	17.43 ± 1.06	40.31 ± 0.91	151.17 ± 4.08	17.76 ± 1.21	31.64 ± 1.59	162.22 ± 10.4
341.0883	191	Caffeoyhexose	1.97 ± 0.07	5.72 ± 0.21	5.77 ± 0.35	2 ± 0.11	5.98 ± 0.4	7.16 ± 0.36	3.79 ± 0.18	5.64 ± 0.17	14.39 ± 0.56	3.31 ± 0.15	8.78 ± 0.33	19.4 ± 0.67
341.5296	191.9969	Caffeoyhexose	nd	nd	nd	nd	nd	nd	2.21 ± 0.06	1.82 ± 0.02	1.6 ± 0.02	2.38 ± 0.11	1.02 ± 0.04	nd
515.1341	353/191	3,5-di-O-Caffeoylquinic acid	4.89 ± 0.09	3.21 ± 0.04	1.32 ± 0.09	4.69 ± 0.13	4.01 ± 0.23	2.15 ± 0.13	3.56 ± 0.19	3.49 ± 0.21	3.04 ± 0.04	3.95 ± 0.13	2.91 ± 0.07	2.44 ± 0.15
647.1752	476.0955/191.0044	Trigalloyl quinic acid	23.25 ± 0.45	39.46 ± 2.23	43.22 ± 1.61	22.91 ± 0.5	42.58 ± 0.53	55.16 ± 1.56	29.09 ± 1.09	32.69 ± 2.05	45.36 ± 2.04	29.79 ± 0.97	45.94 ± 2.05	76.08 ± 4.07
735.1780		Triacyl-O-caffeoyl-O-feruloyl-O-sinapoylquinic acid	2.46 ± 0.08	0.98 ± 0.03	0.34 ± 0.01	2.3 ± 0.14	0.85 ± 0.03	nd	2.11 ± 0.04	0.19 ± 0.01	nd	2.03 ± 0.04	0.24 ± 0.01	nd
515.0966	353/191	3,5-di-O-Caffeoylquinic acid	2.67 ± 0.13	1.99 ± 0.14	0.95 ± 0.03	2.54 ± 0.04	1.57 ± 0.05	0.82 ± 0.02	5.07 ± 0.3	3.69 ± 0.17	2.31 ± 0.11	5.49 ± 0.06	4.8 ± 0.09	4.1 ± 0.08
341.0818	191.007	Caffeoyhexose	0.75 ± 0.03	nd	nd	0.82 ± 0.04	nd	nd	0.58 ± 0.02	0.49 ± 0.03	nd	0.52 ± 0.01	0.38 ± 0.01	nd
295.0038	191.0038	Caffeoyl malate	1.29 ± 0.02	1.15 ± 0.05	1.11 ± 0.06	1.25 ± 0.03	1.02 ± 0.04	0.94 ± 0.05	1.01 ± 0.04	0.99 ± 0.06	0.71 ± 0.02	1.11 ± 0.04	0.94 ± 0.05	0.36 ± 0.01
295.0878	179	Caffeoyl-l-malic acid	3.42 ± 0.19	2.5 ± 0.04	2 ± 0.1	3.36 ± 0.18	2.87 ± 0.13	2.14 ± 0.03	4.19 ± 0.21	2.71 ± 0.16	1.03 ± 0.03	3.94 ± 0.04	3.51 ± 0.05	3.17 ± 0.21
341.0858	191	Caffeoyhexose	13.36 ± 0.39	0.28 ± 0	nd	14.09 ± 0.53	1.58 ± 0.06	nd	27.32 ± 0.96	3.92 ± 0.17	1.05 ± 0.07	25.83 ± 1.43	9.45 ± 0.49	nd
341.0859	192	Caffeoyhexose	0.26 ± 0.02	0.96 ± 0.05	1.03 ± 0.01	0.19 ± 0.01	1.23 ± 0.07	1.49 ± 0.09	0.08 ± 0	0.49 ± 0.01	1.36 ± 0.08	nd	0.12 ± 0	3.57 ± 0.17
315.0476	152/108	Protocatechuic acid hexoside	1.43 ± 0.06	1.04 ± 0.02	1.02 ± 0.03	1.39 ± 0.08	1.08 ± 0.07	0.86 ± 0.06	1.22 ± 0.08	1 ± 0.04	0.65 ± 0.02	1.39 ± 0.09	1.1 ± 0.01	0.29 ± 0
341.0839	191.0015	Caffeoyhexose	2.27 ± 0.13	2.03 ± 0.1	1.43 ± 0.03	2.45 ± 0.06	1.85 ± 0.08	1.53 ± 0.09	8.68 ± 0.6	6.58 ± 0.34	4.47 ± 0.1	8.47 ± 0.43	6.7 ± 0.42	5.8 ± 0.06
635.0353	483.1754/465.2894/313.0659	Trigalloylhexose	23.75 ± 1.33	21.7 ± 0.24	21.29 ± 1.46	24.16 ± 0.29	20.09 ± 1.41	15.22 ± 0.64	41.46 ± 1.77	36.17 ± 1.27	35.01 ± 1.2	43.76 ± 2.66	36.81 ± 1.55	33.97 ± 0.75
371.0693	343/209/191	Hydroxyferulic acid hexoside	1.08 ± 0.07	0.84 ± 0.04	0.54 ± 0.04	0.98 ± 0.03	0.76 ± 0.01	0.62 ± 0.03	2.04 ± 0.07	0.91 ± 0.02	0.74 ± 0.04	2.14 ± 0.09	1.65 ± 0.03	0.66 ± 0.05
353.0657	191/179	Caffeoylquinic acid	7.11 ± 0.38	6.99 ± 0.1	6.3 ± 0.11	7.25 ± 0.29	7.01 ± 0.22	6.63 ± 0.36	nd	nd	nd	nd	nd	nd

353.0671	191/179/163	Caffeoylquinic acid	9.09 ± 0.64	6.68 ± 0.1	3.8 ± 0.05	8.99 ± 0.31	7.58 ± 0.49	5.89 ± 0.27	5.05 ± 0.19	4.75 ± 0.26	2.99 ± 0.14	5.25 ± 0.14	4.44 ± 0.29	3.26 ± 0.15
341.0660	179/161	Caffeoyhexose	5.94 ± 0.14	5.66 ± 0.36	3.7 ± 0.1	5.89 ± 0.12	5.78 ± 0.1	4.12 ± 0.1	nd	nd	nd	nd	nd	nd
341.066	179.0144/161.0085	Caffeoyhexose	1.76 ± 0.07	1.24 ± 0.05	0.72 ± 0.03	1.65 ± 0.05	1.59 ± 0.03	1.4 ± 0.08	3.87 ± 0.04	2.21 ± 0.03	1.18 ± 0.04	3.35 ± 0.12	1.25 ± 0.05	0.69 ± 0.04
337.0640	162	3-Coumaroylquinic acid	2.78 ± 0.18	2.41 ± 0.09	2.01 ± 0.11	2.83 ± 0.08	2.56 ± 0.17	1.59 ± 0.03	1.69 ± 0.05	1.88 ± 0.1	3.68 ± 0.17	1.73 ± 0.03	1.76 ± 0.12	2.98 ± 0.08
179.0198	162	Caffeic acid	2.02 ± 0.09	1.82 ± 0.11	1.48 ± 0.1	1.98 ± 0.05	1.75 ± 0.11	1.26 ± 0.05	2.63 ± 0.11	2.07 ± 0.14	1.87 ± 0.12	2.21 ± 0.08	1.63 ± 0.06	0.98 ± 0.05
371.0743	343/191	Hydroxyferulic acid hexoside	1.57 ± 0.08	1.43 ± 0.04	1.36 ± 0.09	1.65 ± 0.05	1.42 ± 0.07	1.36 ± 0.07	1.29 ± 0.05	1.24 ± 0.03	0.99 ± 0.05	1.25 ± 0.02	1.17 ± 0.06	1.11 ± 0.02
325.0715	163	p-Coumaric acid hexoside	0.2 ± 0.01	0.15 ± 0.01	0.15 ± 0	0.25 ± 0.01	0.21 ± 0.01	0.19 ± 0	nd	nd	nd	nd	nd	nd
325.0602	193.0186	Ferulic acid derivative	nd	nd	nd	nd	nd	nd	2.01 ± 0.05	3.91 ± 0.04	7.78 ± 0.09	4.79 ± 0.13	6.63 ± 0.17	8.08 ± 0.46
367.0741	193	3-Feruloylquinic acid	1.59 ± 0.02	1.5 ± 0.02	1.08 ± 0.03	1.63 ± 0.02	1.45 ± 0.02	1.22 ± 0.08	3.51 ± 0.09	2.91 ± 0.06	2.16 ± 0.03	3.44 ± 0.09	1.71 ± 0.04	nd
515.2644	179.3219/169.1117	Dicaffeoylquinic acid	5.72 ± 0.29	5.73 ± 0.34	5.97 ± 0.17	5.65 ± 0.08	5.69 ± 0.25	6.02 ± 0.13	6.53 ± 0.3	7.16 ± 0.33	7.71 ± 0.33	6.37 ± 0.32	6.77 ± 0.47	7 ± 0.49
387.1422	247.8550/163.0274	3,4-dimethoxycinnamic acid derivative	1.75 ± 0.06	6.79 ± 0.11	13.77 ± 0.36	1.69 ± 0.1	7.12 ± 0.26	15.59 ± 1.01	1.83 ± 0.08	4.86 ± 0.27	12.35 ± 0.64	1.63 ± 0.03	6.64 ± 0.17	15.59 ± 0.17
373.1267	197/182	Syringic acid derivative	1.93 ± 0.08	2.09 ± 0.06	2.54 ± 0.06	1.89 ± 0.13	2.55 ± 0.07	2.98 ± 0.06	2.48 ± 0.03	3.89 ± 0.16	4.62 ± 0.2	2.5 ± 0.04	4.04 ± 0.05	6.13 ± 0.21
387.1356	207.0210/163.0630	3,4-dimethoxycinnamic acid derivative	3.49 ± 0.2	2.93 ± 0.06	2.08 ± 0.13	3.45 ± 0.1	2.99 ± 0.13	2.35 ± 0.12	7.95 ± 0.14	4.99 ± 0.11	4.51 ± 0.29	7.83 ± 0.37	5.12 ± 0.08	3.83 ± 0.23
401.1217	193	Ferulic acid derivative	0.7 ± 0.02	0.59 ± 0.03	0.43 ± 0.03	0.73 ± 0.02	0.65 ± 0.01	0.58 ± 0.01	1.37 ± 0.04	1.19 ± 0.04	0.47 ± 0.01	1.38 ± 0.04	1.06 ± 0.06	nd
387.1437	247/163	3,4-dimethoxycinnamic acid derivative	4.7 ± 0.16	3.69 ± 0.2	3.22 ± 0.12	4.62 ± 0.23	4.02 ± 0.25	3.69 ± 0.17	7.55 ± 0.24	5.33 ± 0.2	3 ± 0.04	7.35 ± 0.37	4.38 ± 0.3	3.71 ± 0.17
385.0975	223	Sinapic acid hexoside	1.51 ± 0.08	1.75 ± 0.03	2.13 ± 0.12	1.5 ± 0.07	1.59 ± 0.02	2.02 ± 0.07	3.05 ± 0.19	3.69 ± 0.22	5.17 ± 0.17	3.34 ± 0.12	3.83 ± 0.26	4.99 ± 0.31
361.1451	209/194/150	Hydroxyferulic acid derivatives	1.23 ± 0.06	1.39 ± 0.02	2.41 ± 0.11	1.2 ± 0.03	1.45 ± 0.06	2.15 ± 0.14	0.87 ± 0.04	2.17 ± 0.1	2.58 ± 0.16	0.92 ± 0.01	1.5 ± 0.05	2.66 ± 0.17
373.1646	197	Syringic acid derivative	0.12 ± 0	0.33 ± 0.02	0.48 ± 0.03	0.1 ± 0	0.29 ± 0.02	0.48 ± 0.01	0.23 ± 0.01	0.34 ± 0.02	0.57 ± 0.04	0.27 ± 0.02	0.35 ± 0.01	0.42 ± 0.02
867.2342		Theaflavin-3,3′-gallate	0.35 ± 0.02	0.48 ± 0.07	0.61 ± 0.10	0.35 ± 0.01	0.51 ± 0.02	0.67 ± 0.01	0.59 ± 0.09	0.6nd.01	0.62 ± 0.02	0.59 ± 0.02	0.61 ± 0.03	0.79 ± 0.02
563.1528	443.1712/315.9880	Theaflavin unknown isomer	0.04 ± 0.00	0.05 ± 0.00	0.06 ± 0.03	0.04 ± 0.00	0.05 ± 0.00	0.06 ± 0.00	0.07 ± 0.00	0.1nd.00	0.01 ± 0.00	0.08 ± 0.00	0.02 ± 0.00	0.01 ± 0.00
1151.3507	867.2342/577.1158. 287.0575	Procyanidin tetramer	30.85 ± 0.83	26.09 ± 0.75	16.01 ± 0.27	30.69 ± 1.3	29.48 ± 1.1	20.48 ± 0.86	46.55 ± 1.17	25.63 ± 0.99	13.11 ± 0.27	53.54 ± 2.74	35.07 ± 1.02	21.66 ± 0.4
315.0867	190.9982	Theaflavoid C.	8.55 ± 0.14	7.98 ± 0.4	7.94 ± 0.28	9.01 ± 0.4	8.56 ± 0.59	7.69 ± 0.25	14.05 ± 0.82	13.68 ± 0.74	10.38 ± 0.49	16.49 ± 0.7	14.97 ± 0.25	11.75 ± 0.58
577.1130	289	B-type procyanidin dimer	16.72 ± 0.45	15.04 ± 0.76	11.62 ± 0.45	16.73 ± 1.07	14.39 ± 0.52	12.58 ± 0.56	25.71 ± 0.3	7.02 ± 0.36	6.32 ± 0.17	27.35 ± 1.62	12.9 ± 0.87	5.22 ± 0.27
305.0412	193	(-)-Epigallocatechin	68.09 ± 1.26	117.66 ± 3.84	122.47 ± 4.26	68.06 ± 3.53	99.58 ± 5.29	135.29 ± 5.1	187.92 ± 11.91	189.15 ± 9.53	351.11 ± 20.05	184.74 ± 2.42	193.5 ± 13.39	411.58 ± 16.05
593.0981	305	(epi)-gallocatechin-(epi)-catechin dimer	28.23 ± 1.67	34.83 ± 1.11	43.41 ± 1.58	28.99 ± 0.79	37.01 ± 0.37	39.58 ± 1.17	70.78 ± 4	81.61 ± 4.37	88.58 ± 3.47	66.25 ± 2.14	80.37 ± 2.58	95.69 ± 4.64
915.0839	457.0785/169.1239	(-)-Epigallocatechin gallate	72.7 ± 3.27	187.6 ± 4.9	260.24 ± 8.46	72.89 ± 3.31	201.58 ± 8.69	289.59 ± 19.49	65.61 ± 2.77	177.87 ± 2.56	410.26 ± 8.21	61.78 ± 1.71	148.59 ± 2.76	444.59 ± 30.05
289.0714	245.128	(-)-Epicatechin	15.19 ± 0.99	19.05 ± 0.86	20.96 ± 0.39	15.8 ± 0.59	22.31 ± 0.44	24.18 ± 1.4	20.39 ± 0.55	28.24 ± 1.06	31.14 ± 1.93	20.25 ± 0.77	31.09 ± 1.82	35.99 ± 2.34
441.1726	289.0441	(+)-Epicatechin gallate	15.82 ± 0.83	16.4 ± 0.6	17.02 ± 0.95	15.63 ± 0.3	17.78 ± 1.03	20.22 ± 1.01	15.02 ± 0.55	15.23 ± 0.55	24.16 ± 1.15	15.21 ± 0.67	18.66 ± 0.33	2.93 ± 0.09
463.0657	301.0958	Peonidin-3-glucoside	nd	nd	nd	nd	nd	nd	0.53 ± 0.03	0.49 ± 0.01	0.43 ± 0.02	0.55 ± 0.01	0.46 ± 0.01	0.41 ± 0.03
419.0671	287.0714	Cyanidin-3-xyloside	nd	nd	nd	nd	nd	nd	5.8 ± 0.28	6.02 ± 0.18	6.17 ± 0.31	5.9 ± 0.11	6.77 ± 0.33	7.32 ± 0.45
609.0660	301.0998	Peonidin-3-rutinoside	nd	nd	nd	nd	nd	nd	2.11 ± 0.08	2.19 ± 0.04	2.28 ± 0.05	2.06 ± 0.08	2.91 ± 0.13	2.92 ± 0.09
595.0715	287.05687	Cyanidin-glycoside	nd	nd	nd	nd	nd	nd	11.23 ± 0.4	11.92 ± 0.54	11.93 ± 0.31	11.17 ± 0.51	11.26 ± 0.27	12.02 ± 0.57
595.1875	287.05688	Cyanidin-coumaroyl-glucopyranoside	nd	nd	nd	nd	nd	nd	3.01 ± 0.15	3.04 ± 0.1	3.53 ± 0.05	3.03 ± 0.04	3.49 ± 0.16	3.89 ± 0.26

552.1593	323.0683	Linden glycoside isomer	nd	nd	nd	2.69 ± 0.07	2.99 ± 0.17	3.68 ± 0.08	nd	nd	nd	2.68 ± 0.1	3.9 ± 0.14	4.59 ± 0.32
595.1262	301	Quercetin-3-O-sambubioside	2.39 ± 0.11	3.17 ± 0.06	3.34 ± 0.05	2.49 ± 0.11	3.28 ± 0.08	2.59 ± 0.13	2.49 ± 0.1	3.48 ± 0.1	4.46 ± 0.15	2.51 ± 0.04	3.35 ± 0.16	5 ± 0.13
565.1772	301	Quercetin dipentoside	7.14 ± 0.42	4.69 ± 0.28	4.66 ± 0.23	7.21 ± 0.1	5.59 ± 0.13	4.66 ± 0.27	12.71 ± 0.74	10.8 ± 0.19	10.78 ± 0.29	12.89 ± 0.82	10.86 ± 0.43	9.99 ± 0.64
625.1073	316	Myricetin coumaroyl hexoside	0.13 ± 0.01	0.1 ± 0.01	0.09 ± 0	0.15 ± 0.01	0.11 ± 0	0.1 ± 0	0.32 ± 0.01	0.14 ± 0.01	0.14 ± 0.01	0.35 ± 0.02	0.09 ± 0.01	0.07 ± 0
465.0624	285/259	Taxifolin 3-O-glucoside	0.61 ± 0.03	0.75 ± 0.03	1.66 ± 0.02	0.59 ± 0.03	0.8 ± 0.05	1.29 ± 0.06	2.25 ± 0.07	3.91 ± 0.09	6.91 ± 0.22	2.22 ± 0.1	3.13 ± 0.22	6.14 ± 0.23
609.1183	301	Quercetin 3-O-rutinoside	19.84 ± 1.18	19.61 ± 0.79	12.48 ± 0.33	20.01 ± 1.3	15.55 ± 0.55	10.29 ± 0.25	39.24 ± 2.55	32.13 ± 1.48	12.93 ± 0.84	40.37 ± 2.39	29.42 ± 1.59	25.17 ± 0.73
639.1277	315	Isorhamnetin 3,7-di-O-hexoside	1.6 ± 0.03	0.94 ± 0.05	0.81 ± 0.04	1.78 ± 0.12	1.01 ± 0.01	0.48 ± 0.02	2.38 ± 0.07	2.1 ± 0.07	1.75 ± 0.07	2.44 ± 0.05	1.71 ± 0.04	1.59 ± 0.1
463.0688	301	Quercetin 7-O-glucoside	2.12 ± 0.11	2.58 ± 0.07	4.06 ± 0.25	2.2 ± 0.07	2.78 ± 0.15	2.98 ± 0.09	5.9 ± 0.39	6.46 ± 0.34	6.75 ± 0.19	5.92 ± 0.11	6.3 ± 0.4	6.99 ± 0.28
447.0617	301.581	Quercetin-3-O-rhamnoside	2.39 ± 0.07	1.6 ± 0.05	1.35 ± 0.02	2.45 ± 0.13	1.99 ± 0.06	1.52 ± 0.03	7.77 ± 0.29	3.38 ± 0.2	3.12 ± 0.05	7.64 ± 0.48	4.83 ± 0.33	4.01 ± 0.14
477.1228	315	Isorhamnetin 3-O-galactoside	1.2 ± 0.06	0.81 ± 0.02	0.67 ± 0.05	1.25 ± 0.08	0.95 ± 0.04	0.48 ± 0.01	1.12 ± 0.02	1.01 ± 0.01	0.65 ± 0.02	1.16 ± 0.03	0.53 ± 0.01	0.47 ± 0.01
447.1739	315.0427	Isorhamnetin3-O-arabinofuranoside	nd	nd	nd	nd	nd	nd	nd	nd	3.1 ± 0.12	nd	0.07 ± 0	4.12 ± 0.25
593.1278	285	Kaempferol 3-O-p-coumaroylhexoside unknown isomer	1.29 ± 0.08	0.82 ± 0.02	0.35 ± 0.02	1.25 ± 0.04	0.99 ± 0.05	0.52 ± 0.01	4.35 ± 0.22	3.08 ± 0.21	2.69 ± 0.06	4.25 ± 0.29	3.61 ± 0.12	2.88 ± 0.09
623.1340	315	Isorhamnetin 3-O-rutinoside	18.93 ± 0.41	18.13 ± 1.22	9.02 ± 0.31	19.01 ± 0.19	16.65 ± 0.79	11.33 ± 0.61	14.18 ± 0.61	11.01 ± 0.58	4.87 ± 0.31	14.35 ± 0.22	8.75 ± 0.41	4.61 ± 0.25
653.1373	315.0298	Myricetin glucosude-glucuronide	0.09 ± 0	0.08 ± 0.01	0.07 ± 0	0.1 ± 0.01	0.09 ± 0	0.08 ± 0	0.85 ± 0.01	0.67 ± 0.02	0.52 ± 0.03	0.91 ± 0.02	0.87 ± 0.04	0.42 ± 0.01
477.0736	315	Isorhamnetin 3-O-glucoside	2.8 ± 0.05	1.14 ± 0.08	1.14 ± 0.06	2.74 ± 0.13	2.08 ± 0.04	1.56 ± 0.02	3.13 ± 0.17	1.46 ± 0.02	1.14 ± 0.04	3.19 ± 0.2	2.75 ± 0.04	1.35 ± 0.09
609.1254	301.0036	Quercetin 3-O-neohesperidoside	0.35 ± 0.02	0.42 ± 0.02	0.49 ± 0.02	0.33 ± 0.02	0.45 ± 0.01	0.59 ± 0.04	0.98 ± 0.03	1.25 ± 0.05	1.37 ± 0.03	0.91 ± 0.03	1.21 ± 0.06	1.42 ± 0.07
		Total	777.24 ± 27.52	940.02 ± 36.26	980.92 ± 32.88	781.39± 30.51	956.82 ± 38.24	1074.94 ± 47.56	1214.33 ± 47.75	1223.64 ± 39.79	1677.51 ± 66.1	1218.34 ± 52.65	1239.34 ± 52.81	1858.43 ± 87.21

Means of three separate analyses ± standard deviation. Abbreviations: WGJ_S – white gooseberry juice with sucrose; WGJ_H – white gooseberry juice with honey; RGJ_S – red gooseberry juice with sucrose; RGJ_H – red gooseberry juice with honey; D0 – day 0 of fermentation; D5 – day 5 of fermentation; D10 – day 10 of fermentation; nd – not detected

Table S2. Changes in individual volatile compounds in white gooseberry-based beverages during SCOBY fermentation (ng/mL).

	WGJ_S			WGJ_H		
	D_0	D_5	D_10	D_0	D_5	D_10
**Esters**						
Acetic acid ethyl ester	185.54 ± 1.97	123.11 ± 1.13	121.85 ± 2.87	164.64 ± 7.56	162.63 ± 8.98	161.59 ± 5.39
Butanoic acid, methyl ester	nd	nd	nd	25.01 ± 0.77	24.70 ± 1.59	24.24 ± 0.59
Acetic acid, octyl ester	386.11 ± 6.66	258.80 ± 15.41	260.71 ± 5.08	88.00 ± 1.51	86.45 ± 1.18	86.13 ± 1.53
Propanoic acid, 2-methyl-, octyl ester	152.78 ± 4.22	102.48 ± 6.88	101.44 ± 1.64	nd	nd	nd
Butanoic acid, 3-methyl-, octyl ester	20.35 ± 1.56	13.63 ± 2.74	13.66 ± 1.49	nd	nd	nd
Ethyl dodecanoate	150.23 ± 4.14	100.44 ± 7.21	99.41 ± 1.75	nd	nd	nd
**Alcohols**						
1-Pentanol	19.93 ± 2.31	12.67 ± 1.32	12.16 ± 0.84	129.16 ± 3.35	128.62 ± 3.52	127.73 ± 3.54
1-Hexen-3-ol	nd	nd	nd	40.40 ± 1.17	40.55 ± 2.50	39.53 ± 1.10
3-Hexen-1-ol, (3Z)-	114.13 ± 1.00	75.70 ± 2.23	75.62 ± 0.65	310.83 ± 3.90	307.12 ± 2.17	306.38 ± 2.44
2-Hexen-1-ol, (2E)-	832.64 ± 2.14	558.92 ± 13.77	559.18 ± 3.63	2859.62 ± 33.89	2864.58 ± 69.02	2850.34 ± 29.54
1-Hexanol	481.00 ± 3.01	323.61 ± 9.27	323.68 ± 3.22	nd	nd	nd
2-Heptanol, 6-methyl-	nd	nd	nd	20.54 ± 0.03	20.28 ± 1.16	20.41 ± 0.68
1-Hepten-3-ol	148.74 ± 2.69	98.94 ± 6.48	98.43 ± 0.29	414.82 ± 4.05	414.43 ± 2.73	411.69 ± 3.29
1-Hexanol, 2-ethyl-	38.34 ± 4.55	25.34 ± 2.49	24.84 ± 0.82	nd	nd	nd
1-Octanol	608.70 ± 10.82	407.30 ± 15.61	407.44 ± 1.72	46.84 ± 0.68	46.26 ± 2.61	45.26 ± 1.70
Fenchyl alcohol	89.13 ± 1.95	59.86 ± 4.23	58.31 ± 1.99	82.76 ± 1.08	81.80 ± 3.66	80.95 ± 2.98
Borneol	69.28 ± 1.23	46.17 ± 3.10	45.13 ± 1.21	96.86 ± 1.66	97.06 ± 3.49	95.69 ± 1.00
Terpinen-4-ol	13.73 ± 1.00	9.61 ± 1.55	9.11 ± 1.00	228.51 ± 2.00	226.74 ± 4.15	226.52 ± 1.29
a-Terpineol	252.15 ± 3.99	169.53 ± 8.97	169.87 ± 4.55	63.51 ± 0.97	63.39 ± 3.14	63.16 ± 0.48
**Acids**						
Propanoic acid, 2-methyl-	nd	nd	nd	12.81 ± 0.74	13.28 ± 1.22	12.73 ± 1.07
Nonanoic acid, 4-methyl-	597.05 ± 8.55	399.68 ± 15.58	398.82 ± 1.34	nd	nd	nd
**Aldehydes**						
Hexanal	24.45 ± 0.58	16.21 ± 1.72	16.75 ± 1.08	91.18 ± 0.75	90.18 ± 1.53	89.30 ± 1.69
Octanal	nd	nd	nd	57.15 ± 0.71	56.51 ± 1.34	56.10 ± 2.25
Nonanal	63.16 ± 1.44	42.09 ± 3.43	42.04 ± 2.87	126.42 ± 1.71	125.61 ± 4.51	123.04 ± 4.04

Cyclomyral	33.59 ± 1.76	22.28 ± 2.74	21.79 ± 1.00	nd	nd	nd
**Ketones**						
6-methyl-Hept-5-en-2-one	nd	nd	nd	66.09 ± 0.77	65.95 ± 2.80	65.72 ± 0.32
Isophorone	3.87 ± 0.09	2.59 ± 0.17	2.59 ± 0.08	nd	nd	nd
**Furans / furan derivatives**						
Furfural	nd	nd	nd	168.87 ± 1.29	167.78 ± 1.55	167.87 ± 1.44
Furan, 2-pentyl-	nd	nd	nd	320.46 ± 4.00	317.95 ± 2.88	316.55 ± 1.65
**Terpenes and terpenoids**						
α-Pinene	nd	nd	nd	14.10 ± 0.62	13.87 ± 2.00	14.01 ± 1.00
Camphene	nd	nd	nd	36.53 ± 1.50	36.71 ± 2.82	36.36 ± 0.42
Limonene	nd	nd	nd	261.10 ± 5.10	257.73 ± 7.95	258.93 ± 4.95
1,4-Cineole	nd	nd	nd	35.92 ± 0.52	35.43 ± 2.99	35.08 ± 0.48
Eucalyptol	13.73 ± 1.00	9.61 ± 1.55	9.11 ± 1.00	79.58 ± 0.34	78.65 ± 3.31	77.78 ± 2.33
cis-Linalool oxide	nd	nd	nd	248.37 ± 1.73	247.71 ± 3.76	246.32 ± 0.21
trans-Linalool oxide	38.34 ± 4.55	25.34 ± 2.49	24.84 ± 0.82	129.60 ± 1.73	128.87 ± 3.22	128.28 ± 0.32
Linalool	118.66 ± 3.16	79.13 ± 5.76	79.12 ± 1.47	nd	nd	nd
δ-Elemene	72.30 ± 2.58	48.15 ± 4.45	47.12 ± 2.59	nd	nd	nd
α-Copaene	18.32 ± 0.82	12.13 ± 2.05	12.16 ± 1.04	nd	nd	nd
(E)-Caryophyllene	710.55 ± 13.74	470.96 ± 28.18	472.25 ± 6.43	nd	nd	nd
α-Humulene	23.41 ± 1.44	15.67 ± 2.57	15.69 ± 1.37	nd	nd	nd
**Total**	5280.2 ± 25.15	3529.91 ± 36.21	3523.15 ± 29.74	6219.65 ± 39.99	6200.81 ± 45.02	6167.69 ± 37.42

Means of three separate analyses ± standard deviation. Abbreviations: WGJ_S – white gooseberry juice with sucrose; WGJ_H – white gooseberry juice with honey; RGJ_S – red gooseberry juice with sucrose; RGJ_H – red gooseberry juice with honey; D0 – day 0 of fermentation; D5 – day 5 of fermentation; D10 – day 10 of fermentation; nd – not detected

Table S3. Changes in individual volatile compounds in red gooseberry-based beverages during SCOBY fermentation (ng/mL).

	RGJ_S			RGJ_H		
	D_0	D_5	D_10	D_0	D_5	D_10
**Esters**						
Acetic acid ethyl ester	555.1 ± 16.88	841.37 ± 282.67	837.44 ± 281.35	733.16 ± 23.25	722.61 ± 21.43	720.89 ± 22.81
Butanoic acid, ethyl ester	5.91 ± 1.27	8.88 ± 2.98	7.59 ± 2.55	55.28 ± 2.36	54.25 ± 6.28	56.92 ± 2.67
Hexanoic acid, ethyl ester	13.92 ± 1.35	21.57 ± 7.25	21.51 ± 7.23	69.86 ± 3.56	69.53 ± 8.94	68.57 ± 5.38
Octanoic acid, ethyl ester	93.64 ± 5.96	144.67 ± 48.60	141.68 ± 47.60	148.94 ± 7.13	151.27 ± 17.75	146.25 ± 8.63
Decanoic acid, ethyl ester	50.19 ± 3.01	77.41 ± 26.01	75.9 ± 25.50	nd	nd	nd
Dodecanoic acid, ethyl ester,	107.14 ± 6.78	164.97 ± 55.42	160.66 ± 53.98	nd	nd	nd
Tetradecanoic acid, ethyl ester	35.01 ± 2.98	54.57 ± 18.33	53.13 ± 17.85	nd	nd	nd
Hexadecanoic acid, ethyl ester	22.36 ± 1.41	35.53 ± 11.94	34.16 ± 11.48	nd	nd	nd
1-Butanol, 3-methyl-, acetate	10.54 ± 0.56	16.50 ± 5.54	13.92 ± 4.68	nd	nd	nd
1-Butanol, 2-methyl-, acetate	2.95 ± 0.06	5.08 ± 1.71	3.80 ± 1.28	nd	nd	nd
**Alcohols**						
1-Propanol, 2-methyl-	nd	nd	nd	304.78 ± 16.71	305.58 ± 19.93	304.14 ± 8.99
1-Butanol, 3-methyl-	245.07 ± 6.61	374.37 ± 125.77	368.12 ± 123.68	4897.88 ± 109.49	4865.49 ± 42.59	4897.69 ± 34.52
1-Butanol, 2-methyl,	112.2 ± 5.30	164.97 ± 55.42	169.51 ± 56.97	nd	nd	nd
1-Pentanol	nd	nd	nd	59.11 ± 2.20	58.83 ± 6.30	58.76 ± 6.32
1-Hexanol	nd	nd	nd	107.48 ± 4.73	104.67 ± 11.27	102.89 ± 6.92
1-Octanol	nd	nd	nd	84.45 ± 4.70	84.8 ± 11.02	82.43 ± 6.48
1-Octen-3-ol	nd	nd	nd	290.19 ± 4.15	291.05 ± 16.79	289.2 ± 6.62
1-Hexanol, 2-ethyl-	7.59 ± 0.79	11.42 ± 3.84	11.39 ± 3.83	nd	nd	nd
Phenethyl alcohol	nd	nd	nd	648.70 ± 18.94	639.31 ± 17.82	655.70 ± 19.46
**Acids**						
Butanoic acid, 3-methyl-	75,08	115.48 ± 38.80	112.59 ± 37.83	nd	nd	nd
**Aldehydes**	nd	nd	nd	nd	nd	nd
Benzaldehyde	nd	nd	nd	224.17 ± 9.64	222.31 ± 20.02	218.64 ± 10.86
n-Octanal,	26.99 ± 2.69	43.15 ± 14.50	40.48 ± 13.60	nd	nd	nd
Nonanal	49.77 ± 3.52	77.41 ± 26.01	74.64 ± 25.08	99.8 ± 4.72	101.61 ± 13.68	96.99 ± 10.05
Cyclomyral	38.81 ± 3.44	60.91 ± 20.46	58.19 ± 19.55	nd	nd	nd

**Ketones**						
Furan, 2-pentyl-	nd	nd	nd	681.71 ± 22.03	675.95 ± 28.75	686.57 ± 24.21
**Terpenes & terpenoids**						
α-Pinene	14.76 ± 1.36	22.84 ± 7.67	22.77 ± 7.65	36.08 ± 3.54	36.68 ± 7.17	36.01 ± 3.63
Camphene	30.79 ± 2.18	48.22 ± 16.20	46.81 ± 15.73	62.95 ± 4.67	63.42 ± 8.54	61.21 ± 7.31
Myrcene	182.64 ± 12.22	279.19 ± 93.80	273.24 ± 91.80	0	0	0
p-Cymene	12.23 ± 1.35	19.04 ± 6.40	18.98 ± 6.38	38.39 ± 3.61	38.97 ± 7.47	36.62 ± 4.94
cis-Linalool oxide	7.59 ± 0.79	12.69 ± 4.26	11.39 ± 3.83	0	0	0
trans-Linalool oxide	9.7 ± 1.32	15.23 ± 5.12	15.18 ± 5.10	0	0	0
Linalool	33.32 ± 2.87	52.03 ± 17.48	50.60 ± 17.00	0	0	0
**Total**	1743.33	2667.51	2623.66	8542.93	8486.33	8519.45

Means of three separate analyses ± standard deviation. Abbreviations: WGJ_S – white gooseberry juice with sucrose; WGJ_H – white gooseberry juice with honey; RGJ_S – red gooseberry juice with sucrose; RGJ_H – red gooseberry juice with honey; D0 – day 0 of fermentation; D5 – day 5 of fermentation; D10 – day 10 of fermentation; nd – not detected
